# Cochlear implantation in adults with acquired single-sided deafness improves cortical processing and comprehension of speech presented to the non-implanted ears: a longitudinal EEG study

**DOI:** 10.1093/braincomms/fcaf001

**Published:** 2025-01-03

**Authors:** Ya-Ping Chen, Patrick Neff, Sabine Leske, Daniel D E Wong, Nicole Peter, Jonas Obleser, Tobias Kleinjung, Andrew Dimitrijevic, Sarang S Dalal, Nathan Weisz

**Affiliations:** Centre for Cognitive Neuroscience, University of Salzburg, 5020 Salzburg, Austria; Department of Psychology, University of Salzburg, 5020 Salzburg, Austria; Centre for Cognitive Neuroscience, University of Salzburg, 5020 Salzburg, Austria; Department of Otorhinolaryngology, Head and Neck Surgery, University Hospital Zurich, University of Zurich, 8091 Zurich, Switzerland; Department of Psychiatry and Psychotherapy, University of Regensburg, 93053 Regensburg, Germany; Neuro-X Institute, Ecole Polytechnique Fédérale de Lausanne (EPFL), Campus Biotech, 1202 Geneva, Switzerland; RITMO Centre for Interdisciplinary Studies in Rhythm, Time and Motion, University of Oslo, 0313 Oslo, Norway; Department of Musicology, University of Oslo, 0313 Oslo, Norway; Department of Neuropsychology, Helgeland Hospital, 8657 Mosjøen, Norway; Department of Psychology, Universität Konstanz, 78457 Konstanz, Germany; Department of Psychology, Universität Konstanz, 78457 Konstanz, Germany; Department of Otorhinolaryngology, Head and Neck Surgery, University Hospital Zurich, University of Zurich, 8091 Zurich, Switzerland; Center of Brain, Behavior, and Metabolism, University of Lübeck, 23562 Lübeck, Germany; Department of Psychology, University of Lübeck, 23562 Lübeck, Germany; Department of Otorhinolaryngology, Head and Neck Surgery, University Hospital Zurich, University of Zurich, 8091 Zurich, Switzerland; Evaluative Clinical Sciences Platform, Sunnybrook Research Institute, Toronto, ON M4N 3M5, Canada; Otolaryngology-Head and Neck Surgery, Sunnybrook Health Sciences Centre, Toronto, ON M4N 3M5, Canada; Faculty of Medicine, Otolaryngology-Head and Neck Surgery, University of Toronto, Toronto, ON M5S 3H2, Canada; Department of Psychology, Universität Konstanz, 78457 Konstanz, Germany; Department of Clinical Medicine, Center of Functionally Integrative Neuroscience, Aarhus University, 8200 Aarhus, Denmark; Centre for Cognitive Neuroscience, University of Salzburg, 5020 Salzburg, Austria; Department of Psychology, University of Salzburg, 5020 Salzburg, Austria; Neuroscience Institute, Christian Doppler University Hospital, Paracelsus Medical University, 5020 Salzburg, Austria

**Keywords:** cochlear implant, EEG, single-sided deafness, speech comprehension, representational similarity analysis

## Abstract

Former studies have established that individuals with a cochlear implant (CI) for treating single-sided deafness experience improved speech processing after implantation. However, it is not clear how each ear contributes separately to improve speech perception over time at the behavioural and neural level. In this longitudinal EEG study with four different time points, we measured neural activity in response to various temporally and spectrally degraded spoken words presented monaurally to the CI and non-CI ears (5 left and 5 right ears) in 10 single-sided CI users and 10 age- and sex-matched individuals with normal hearing. Subjective comprehension ratings for each word were also recorded. Data from single-sided CI participants were collected pre-CI implantation, and at 3, 6 and 12 months after implantation. We conducted a time-resolved representational similarity analysis on the EEG data to quantify whether and how neural patterns became more similar to those of normal hearing individuals. At 6 months after implantation, the speech comprehension ratings for the degraded words improved in both ears. Notably, the improvement was more pronounced for the non-CI ears than the CI ears. Furthermore, the enhancement in the non-CI ears was paralleled by increased similarity to neural representational patterns of the normal hearing control group. The maximum of this effect coincided with peak decoding accuracy for spoken-word comprehension (600–1200 ms after stimulus onset). The present data demonstrate that cortical processing gradually normalizes within months after CI implantation for speech presented to the non-CI ear. CI enables the deaf ear to provide afferent input, which, according to our results, complements the input of the non-CI ear, gradually improving its function. These novel findings underscore the feasibility of tracking neural recovery after auditory input restoration using advanced multivariate analysis methods, such as representational similarity analysis.

## Teaser

Chen *et al*. report that cochlear implant users with single-sided deafness show neural pattern normalization after implantation, particularly in the ear without the cochlear implant. Using representational similarity analysis, they observed increased neural similarity to normal hearing individuals and peak decoding accuracy for speech comprehension between 600 and 1200 ms post-stimulus.

## Graphical Abstract

**Graphical Abstract:**
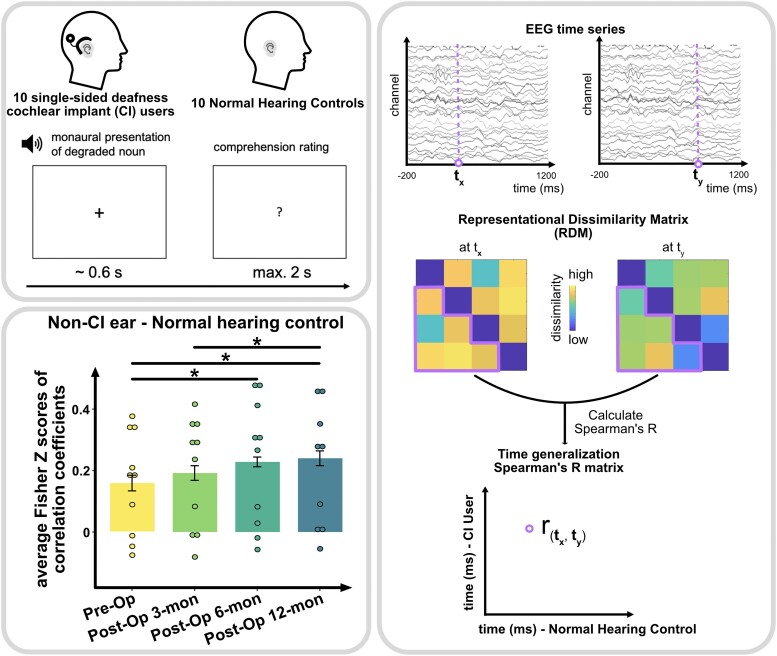

## Introduction

Single-sided deafness (SSD) is a condition in which an individual has normal hearing thresholds in one ear and severe-to-profound hearing loss in the other. This condition can significantly impact an individual’s ability to hear in certain situations, such as when sound is coming from the side of the deaf ear. It can also affect an individual’s ability to understand speech-in-noisy environments and their quality of life.^1,2^ Brain activities associated with hearing through the normal hearing side of people with SSD have been reported to be altered compared to individuals with bilateral normal hearing.^3-5^ The global prevalence of SSD is unclear, while there is an estimated prevalence of 0.1–0.5% in the USA.^6,7^ SSD is caused by a variety of factors, including trauma to the ear, infections and genetic predisposition.^8,9^ It is important to note that a combination of these factors can lead to SSD. Treatments for SSD include hearing aids, bone conduction devices and cochlear implants (CIs).^1,10,11^ Diagnostic criteria for SSD typically involve measuring pure-tone hearing thresholds in both ears between 125 and 8000 Hz (in octave or semi-octave steps). The hearing threshold in the hearing loss ear is typically >90 dB at all frequencies. An affected individual can only hear very loud sounds in the SSD ear and may not be able to hear soft or moderate sounds at all. On the other hand, the hearing threshold in the unaffected ear is typically within the normal range (25 dB or less at all frequencies).^12^

Overall, cochlear implantation has brought about positive outcomes for people with SSD. Single-sided CI implantation generally shows beneficial effects of the intervention in both children^13-15^ and adults.^10,16-19^ These positive outcomes include increased performance in directional hearing, improved speech-in-noise performance, enhanced quality of life, reduced listening effort and suppression of tinnitus.^10,13,18,20^ However, the impact of cochlear implantation on the performance of non-CI, clinically healthy ears has not been well characterized.

Neurophysiological data generally support these described behavioural findings, while not all common neuroimaging methods are feasible to study CI outcomes. For example, MRI and magnetoencephalography are not suitable due to the ferromagnetic and electromagnetic properties of a CI device.^21,22^ PET and functional near-infrared spectroscopy have been employed in SSD and CI studies, providing confirmatory results regarding the beneficial effect of CI implantation. However, these modalities lack temporal and, to some extent, spatial resolution and are invasive due to the use of radioactive tracers.^23,24^

Most EEG studies in the context of SSD and CI have employed auditory event-related paradigms and have interpreted classical components of the event-related potentials (ERPs) in SSD children^25-28^ and adults.^29-31^ In most of these studies, early auditory components were investigated, showing the ‘normalization’ of peaks and latencies after CI implantation and/or over time (i.e. N1, P2 peaks and latencies and partly mismatch negativity). Normalization here refers to the process by which the ERPs of CI users become more similar to those of normal hearing controls. This process can involve both increases and decreases in ERP amplitudes and latencies, depending on experimental conditions and ERP components investigated. For example, Finke *et al*.^30^ found that CI users showed greater processing effort and delayed response times compared to normal hearing listeners, but with some normalization in ERP components over time. Another study demonstrated that cortical organization can be restored in young children with SSD, achieving ERP patterns similar to normal hearing children with consistent CI use.^26^ Wedekind *et al*.^31^ observed near-normal higher-order processing in CI users, but with significant variability in ERP measures, still indicating partial normalization. Mixed results are also reported, where functional and neurophysiological improvements were absent or hampered^32^ as not all CI users benefit from the implant in the same manner.

The current consensus on understanding plasticity in the context of CI and SSD remains somewhat unclear, with discussions centred around adaptive and/or cross-modal plasticity.^33-38^ Longitudinal data, especially regarding time courses in the adult population, are scarce, making it challenging to develop a comprehensive understanding of the neuroplastic changes associated with CI intervention. Further research is needed to elucidate the neural mechanisms underlying improvements in speech comprehension and overall rehabilitation outcomes in the adult human population with CI and SSD.

The utilization of machine learning methods, such as multivariate pattern analysis (MVPA) and representational similarity analysis (RSA), may offer several advantages over traditional univariate approaches, such as the aforementioned classical ERP paradigms.^39-41^ MVPA allows us to extract meaningful patterns from complex and multidimensional data and to gain a deeper understanding of how different patterns of neural activity are associated with specific cognitive processes or conditions. MVPA can be applied to tasks such as classification (e.g. assigning a data sample to predefined categories), regression (e.g. predicting continuous values) and decoding (e.g. inferring mental states from brain activity patterns). RSA is a member of the family of MVPA methods and a specific technique for exploring the relationships between patterns of neural activity in response to different stimuli or conditions. This method allows us to compare the similarity of brain patterns between patients and healthy controls in a multivariate way (considering all features) without the need to establish any spatial correspondence for EEG data, as it derives from the direct comparison of measures. In summary, while MVPA excels in distinguishing between states based on complex patterns of brain activity, RSA provides a powerful framework for understanding the underlying representational structure of these brain patterns, making it particularly suitable for studies with high variability or limited sample sizes. Studies have applied RSA to compare patterns of neural activity in visual categorization between infants and adults^42^ and in memory encoding and retrieval between young and older adults.^43^ However, RSA has not been applied to track the effects of CIs yet. We thus consider RSA for comparing CI and normal hearing individuals and ears in several sessions in response to complex manipulated speech stimuli as a very promising approach in tracking the neural patterns elicited by speech during rehabilitation: a gradual improvement of speech processing could be reflected by neural patterns becoming increasingly similar to those observed in normal hearing individuals.

In this study, we primarily focus on post-lingual adult SSD (referred to simply as SSD) and explore the longitudinal neurobehavioral effects on cortical processing performance when speech presents to each ear following CI implantation. To the best of our knowledge, no previous neurophysiological analyses have employed RSA on longitudinal clinical data in the context of SSD and CI. Using RSA analysis allows us to compare time-resolved and time-generalization neural representation patterns with high temporal resolution, utilizing data from all EEG electrodes. Brain activity patterns from normal hearing controls serve as the main reference, assuming normal, unaltered neural speech processing, while non-CI ears and CI ears of implanted SSD patients are compared against the reference category respectively. Importantly, we can track these brain activity patterns over several time points, including pre-CI implantation, 3, 6 and 12 months after CI implantation, enabling us to investigate neuroplastic dynamics triggered by CI implantation over time.

Here, we conducted an EEG study with a sample of 10 individuals with SSD along with 10 normal hearing controls. We presented various temporally and spectrally degraded words monaurally at four measurement time points spanning a year. The utilization of degraded words as stimuli was based on the rationale that acoustic signals of auditory stimuli undergo spectral degradation through the use of CIs. Furthermore, by adding temporal degradation we aimed to further emulate adverse listening conditions based on an established paradigm.^44,45^ Applying RSA, we aimed to investigate the impact of CI on the processing of degraded speech in both CI and non-CI ears. Our findings demonstrate that speech perception improves post-operatively in both ears, with greater benefits observed in the non-CI ear. Importantly, the RSA reveals that speech-related neural activity following stimulation of non-CI ear becomes increasingly similar to that of individuals with normal hearing over the course of the year. Overall, our study suggests that performance benefits of CIs in SSD may be mediated by the improvement in processing of input from the non-CI ear, which remains the more precise source of information even after implantation.

## Materials and methods

Patients were recruited as the part of a multicentre clinical trial with patients from the Departments of Otorhinolaryngology at University Hospital Zurich and University Hospital Bern between 2012 and 2016. Additional details about the study design and clinical outcomes were published in 2019.^46^ This study primarily focuses on the neurobehavioral and neuroplastic effects of CI implantation on degraded speech performance.

### Participants

Ten individuals with SSD were included in this study (mean age = 46.9 years, age range = 27–63 years, 4 females, see Table 1). An age- and sex-matched group with normal hearing was recruited for comparison (mean age = 48.2 years, age range = 29–61 years, 4 females). The mean duration of deafness before CI implantation was 1.66 years (range = 0.8–4.6 years). The ‘clinically healthy’ ears of the SSD individuals and normal hearing controls were defined by pure-tone audiogram measures (see Supplementary Fig. S1). Further audiometric and psychometric details (e.g. pure-tone audiometry, Oldenburg sentence test) are available in our previous clinical article.^46^ Ethics committees of University Hospital Zurich and University Hospital Bern gave ethical approval for this work respectively (reference numbers KEK-ZH 2012–0034 and KEK-BE 233/12).

**Table 1:** Descriptives of SSD participants

CI case #	Sex	Side of deafness	Age at CI activation (years)	Duration of deafness (years)
1	F	L	27	1.0
2	M	R	36	1.7
3	F	L	42	1.6
4	M	R	45	4.6
5	M	R	48	0.8
6	M	L	48	1.5
7	M	L	50	1.5
8	M	L	54	1.2
9	F	R	56	1.3
10	F	R	63	1.4

F, female; L, left; M, male; R, right.

### Stimuli and procedures

For the degraded speech performance test, we selected 216 standard German words from a 560-item pool of recordings of spoken German words.^44,47^ We created audio files with 3 levels of spectral degradation (4, 8 and 16 bands) and 3 levels of temporal envelope smoothing (2, 4 and 8 Hz), resulting in a total of 9 conditions, each consisting of 24 nouns (Supplementary Figs. S2 and S3 and Tables S1.1 and S1.2). From each original, unprocessed audio file, various degraded versions were created using a MATLAB-based noise-band-vocoding algorithm. The MATLAB code used in this study is available in our GitLab repository (see ‘Data/Code availability’). Noise vocoding is an effective technique for manipulating the spectral detail while preserving the temporal envelope of the speech signal,^48^ allowing for graded and controlled intelligibility based on the number of spectral bands used, with more bands yielding a more intelligible speech signal. The extracted amplitude envelope from each band is used to modulate the white noise for that specific band. In noise vocoding, spectral degradation is varied by specifying the number of spectral bands (bandpass filters) to be extracted. The extracted envelopes in each band were smoothed (low-pass filtered) by a value that does not affect intelligibility (e.g. 400 Hz, as relevant temporal perturbations contributing to intelligibility in speech seem to lie around 20 Hz).^49^ In addition to spectral degradation, we systematically varied the temporal smoothing of the amplitude envelopes over a frequency range previously identified to distinctly influence intelligibility. Notably, noise vocoding allows for the orthogonal manipulation of spectral and temporal variations in any given signal. The design was adapted from our two previous studies.^44,45^ Each stimulus was presented monaurally, once in each ear (i.e. the non-CI ear and the CI ear), during two EEG recording sessions on each measurement day. There were four different sequences of the 216 German words and the arrangement of these sequences was applied in a counterbalanced manner across sessions and ears.

Participants rated the comprehension level of each degraded word on a scale of 1 (‘not’) to 4 (‘well’) using a computer keyboard after hearing each word. Subjective scales were chosen for word comprehension as they provide insights into individual subjective comprehension experiences over time. Measures such as multiple choice responses may lack this level of data sensitivity and resolution due to limited measurement time. Furthermore, these methods might be accompanied with potential (increased) risk of learning effects, possibly limiting the validity of the outcomes in this longitudinal design. Stimuli were presented either through earphones (to non-CI ears at 60 dBA SPL via Etymotic ER-2 air-conducting earphones) or through the CI audio input port (Nucleus CI422 implant with Slim Straight electrode; Cochlear Ltd, Australia).

Each trial began with a silent phase lasting 0.5–1.5 s (with jittered length), followed by 3 s with the presentation of the stimulus 0.6 ± 0.12 s (range 0.28–1.04 s). Participants rated the comprehensibility of the stimulus without a time limit when a question mark displayed on the screen, which disappeared after the button was pressed. Finally, there was a 2-s break before the onset of the next trial.

### EEG measurement

EEG recordings took place in an electrically shielded room. A 128-channel ANT-neuro EEG system with a Waveguard cap (ANT Neuro, Enschede, The Netherlands) was used in a standardized montage based on the 10–5 system.^50^ Device-based average reference was used as an online reference, and impedances were kept below 5 kΩ. The recording sampling frequency was set at 2048 Hz, and no online filtering was applied. Two EEG sessions (one session per year) were recorded from the normal hearing participants as reference data. CI participants, on the other hand, underwent seven recording sessions, including one preoperative session and two sessions during each follow-up visit over 12 months (3, 6 and 12 months after CI implantation, respectively).

### EEG data pre-processing

EEG data pre-processing was conducted using MATLAB R2020a (Version 9.8, Mathworks, USA) and Fieldtrip Toolbox^51^ with in-house modifications. First, data for later RSA analysis (including data from both CI users and normal hearing controls) were filtered with a bandpass filter between 0.1 and 80 Hz (finite impulse response filter with Kaiser window) and a discrete Fourier transform filter around 50 Hz to reduce line noise, and then epoched from −1 to 1.5 s. To remove general EEG artefacts (such as eye movement and heartbeats), we applied independent component analysis (ICA). For the ICA, raw data were filtered with a bandpass filter between 1 and 30 Hz (finite impulse response filter with Kaiser Window), downsampled to 500 Hz and epoched from −1 to 1.5 s relative to the word onset. With the Fieldtrip automatic artefact rejection algorithm, we rejected trials with *z*-values higher than 100 before conducting ICA. On average, 4.15 ± 1.41 (SD) components for CI users and 4.15 ± 1.42 (SD) components for normal hearing controls were removed per session across participants. The automatic artefact rejection algorithm was applied once more to eliminate trials with *z*-values exceeding 50.

### Cochlear implant artefact suppression in EEG

To mitigate CI artefacts, we used an ICA method known as second-order blind identification (SOBI).^52,53^ After removing eye movements and cardiac activity, we applied SOBI exclusively to the CI users’ CI ear data. Components displaying spikes at the onset and/or offset of stimuli were identified as CI artefacts and removed. On average, 3.25 ± 1.88 (SD) components were removed per session across participants.

### EEG data analysis

To characterize the temporal dynamics, regions of interest and the shared representation of speech degradation among CI ears, non-CI ears and normal hearing controls, we utilized multivariate time-resolved classification/RSA and relating representations among those datasets on the EEG data of all participants and sessions.^42,54^ Detailed descriptions of these analyses are provided in the following sections. The analyses were performed using MATLAB R2020a (Version 9.8, Mathworks, USA), Fieldtrip Toolbox^51^ and scripts from Xie *et al*.^42^ with in-house modifications. Due to the limited number of trials (24 trials for each condition) for RSA, we pooled two conditions based on the subjective comprehension rating and excluded the condition with the highest accuracy. This resulted in four conditions: ‘very easy’, ‘easy’, ‘difficult’ and ‘very difficult’, on which based on our analysis. Both the CI and normal hearing datasets were separately analysed in an analogous manner on a per-participant basis. We provided conventional ERP analysis as a sanity check for data quality (see Supplementary Text S1 and Fig. S4).

#### Time-resolved classification

**Figure 1:**
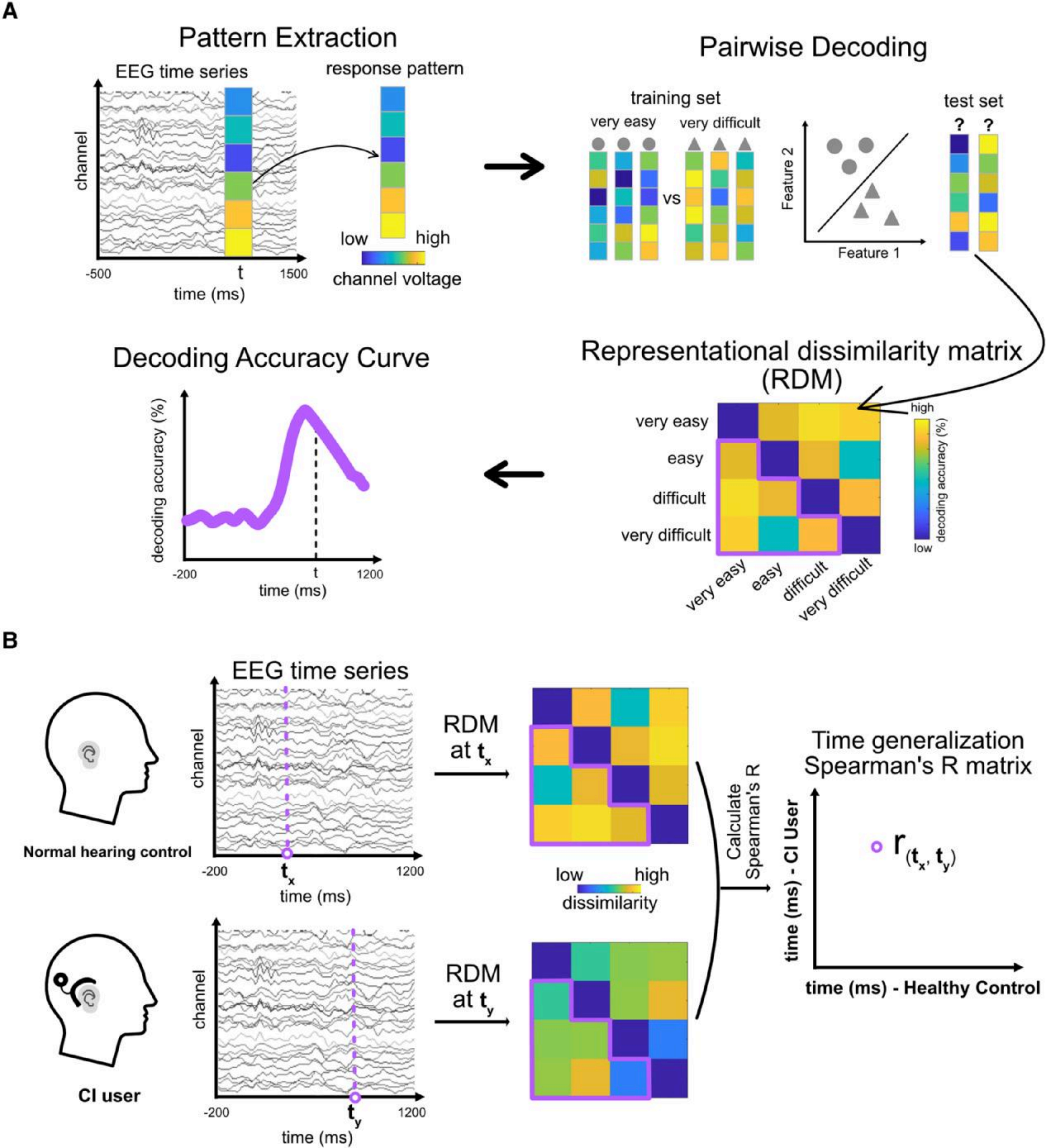
**Time-resolved multivariate analysis on EEG data. (A)** General overview of the RSA. First, we extracted condition-specific EEG sensor values for every time point in every epoch (trial) and formed them into response vectors. Then, using a leave-one-out cross-validation scheme, we trained and tested a SVM to classify speech comprehension from the response vectors. The decoding result (pairwise decoding accuracy, e.g. the very easy condition versus the very difficult condition, 50% chance level) for every time point was aggregated into a RDM of size 4 × 4. Here, decoding accuracy is used as a dissimilarity measure. The matrix is symmetric along the diagonal, which is undefined. Averaging the lower triangular part of the matrix (accuracies within the marked area) resulted in grand average decoding accuracy as an index of how well neural representations distinguish speech comprehension at each time point. Grand average decoding accuracies for each time point were formed into the decoding accuracy curve which depicted how the brain discriminates speech comprehension over time. **(B)** General overview of the shared representation analysis. We employed RDMs to relate speech degradation representations among CI users and normal hearing controls. We computed Spearman’s R to correlate RDMs between CI ears and normal hearing controls as well as between non-CI ears and normal hearing controls for all time point combinations (*t_x_*, *t_y_*).

Time-resolved, within-subject MVPA was applied to the EEG data to identify the temporal dynamics of speech comprehension representations in both CI and normal hearing brains (Fig. 1A). At each time point within the EEG epoch (ranging from −500 to +1500 ms relative to the stimulus onset), trial-specific EEG channel activations were extracted from both CI users and normal hearing control participants (10 each) and were organized into pattern vectors corresponding to the four category conditions: ‘very easy’, ‘easy’, ‘difficult’ and ‘very difficult’ from the stimulus set. To enhance the signal-to-noise ratio (SNR), we randomly distributed raw trials into four bins of approximately equal size and averaged them to create four pseudo-trials.^42,55^ We used a leave-one-pseudo-trial-out cross-validation classification method and trained classifiers using linear support vector machines (SVMs), implemented in the LIBSVM toolbox in MATLAB,^56^ to decode pairwise combinations of any two conditions per session. This procedure was repeated 1000 times, with each iteration involving a new random assignment of trials into four pseudo-trials for each condition. The decoding results (pairwise decoding accuracy, e.g. the very easy condition versus the very difficult condition, with a 50% chance level) at each time point were aggregated into a 4 × 4 representational dissimilarity matrix (RDM). The matrix exhibits symmetry along the diagonal, which remains undefined. By averaging the lower triangular part of the matrix (Fig. 1A right bottom, accuracies within the red line area), we obtained the grand average decoding accuracy, serving as an index of the effectiveness in distinguishing speech comprehension levels of neural representation at each time point. These grand average decoding accuracies for each time point were then used to construct a decoding accuracy curve, illustrating how the brain discriminates speech comprehension over time.

#### Relating degraded speech representations among cochlear implant users and normal hearing controls

To investigate how CI ears, non-CI ears and normal hearing controls share common degraded speech processing representations, we related their degraded speech representations in a representational similarity time-generalization analysis (Fig. 1B). We computed shared representations of two combinations: (i) between non-CI ears and normal hearing controls and (ii) between CI ears and normal hearing controls. To enhance SNR, we averaged the RDMs of the non-CI and CI ear. Subsequently, we correlated the average RDM from the CI users with each normal hearing participant’s RDM across all combinations of time points. This yielded 10 correlation coefficient matrices, with the time points indexed in both rows and columns.

### Statistical analysis

The behavioural comprehension ranking data in response to the variously degraded words were analysed using cumulative link mixed models (CLMMs) in R (version 3.6.2, R Development Core Team, 2019) with the ‘ordinal’ package.^57^ The fixed effects in the CLMMs consisted of the predictors of interest (ear, session and condition), and the random effects included the variables of participant and item. All models were fitted using the maximum likelihood estimation. The best-fitting model was determined through backward stepwise model selection. The outcomes of the final model are reported in the result section. The *P*-values for the fixed effects in the CLMM were obtained via the ‘Anova.clmm’ function from the ‘RVAideMemoire’ package.^58^ The *post hoc* pairwise contrasts for each pair of levels of the group, session and condition variables were calculated using the ‘emmeans’ and ‘pairs’ functions with the ‘emmeans’ package.^59^ The effect size Cohen’s *d* was computed using the ‘cohens_d’ function from the ‘rstatix’ package: *d* = 0.2 indicates a small effect; *d* = 0.5 indicates a medium effect; *d* = 0.8 indicates a large effect.^60^

To test whether the time-resolved decoding accuracies exceeded the chance level (pairwise decoding accuracy, with a 50% chance level), we conducted a non-parametric, cluster-based statistical analysis using the maximum cluster size method.^61^ The cut-off was determined through a sign permutation test based on the distribution of *t*-values from all possible permutations of the measured accuracy values. The cluster threshold was set at 0.05, one-sided. To identify significant clusters, the threshold was also set at 0.05, one-sided.

To examine the correlation coefficient matrices for the shared representation between two groups, we first applied Fisher-z transformation to the correlation coefficients. Subsequently, we expanded the cluster-based approach described above to 2D. The threshold settings were the same as mentioned earlier (*P* < 0.05, one-sided).

To test the correlation coefficient matrices extracted from the shared representation matrices of non-CI ears and normal hearing controls over four sessions, we first Fisher-z transformed and then averaged the values within the 600 and 1200 ms time window, yielding a single value for each participant per session. A permutation repeated measures *F*-test was employed to examine the session effect using the ‘permuco’ package in R.^62^ Additionally, permutation paired *t*-tests were conducted to compare Fisher-z transformed correlation coefficients between any two sessions, with adjusted alpha levels [0.0083 (0.05/6) for the non-CI ears, 0.0167 (0.05/3) for the non-CI ears] applied through Bonferroni correction, utilizing the ‘RVAideMemoire’ package in R.^58^ In the both permutation analysis, 10 000 permutation samples and corresponding test statistics were obtained to generate the permutation distribution.

## Results

### Cochlear implant users’ comprehension rating improved over sessions on both cochlear implant ears and non-cochlear implant ears

**Figure 2:**
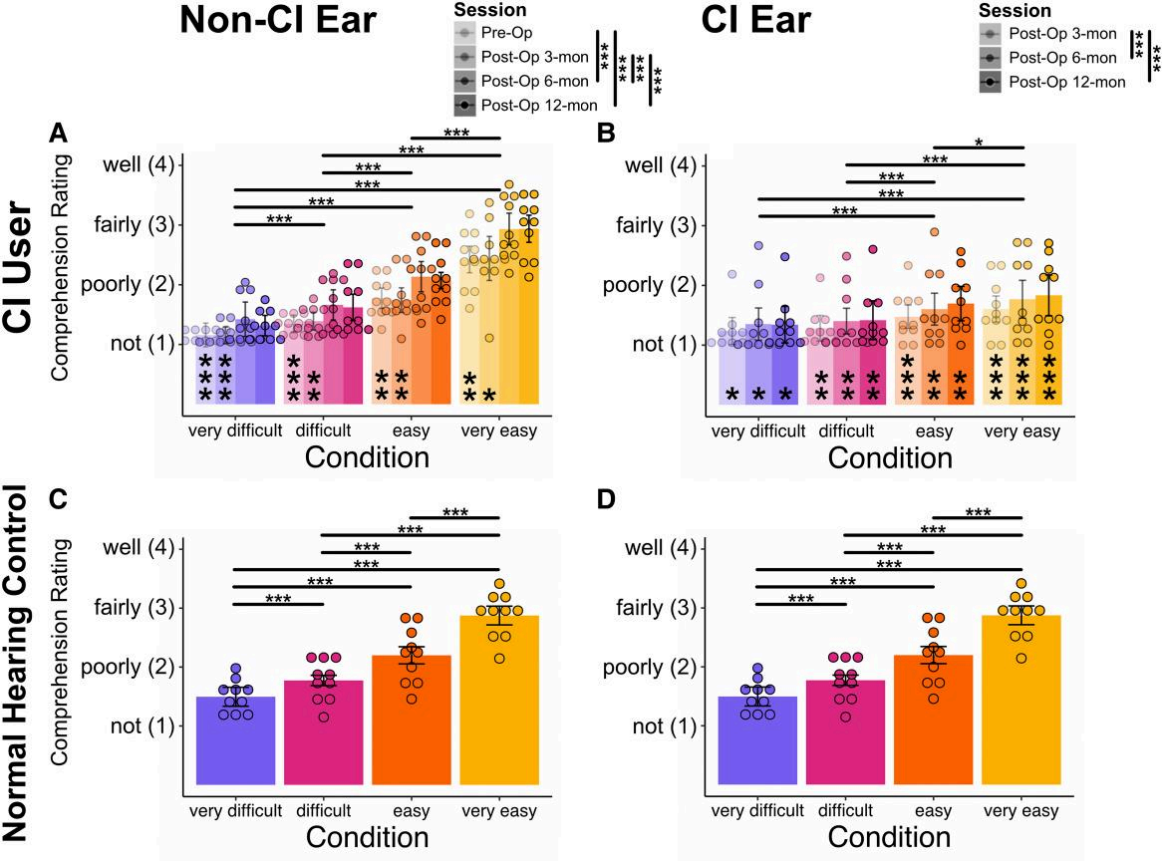
**Results of comprehension ratings. (A**, **B)** The subjective comprehension rating increased over time not only in the CI ear but also in the non-CI ear. The asterisks on the bottom of the bars in the non-CI ear and CI ear denote whether there is a significant difference between those and normal hearing controls. **(A**, **C**, **D)** Significant differences between all pairs of conditions were found in the normal hearing controls and the non-CI ear (*N* = 10 for each group; all *P* < 0.001, CLMMs, pairwise comparisons corrected by Tukey test). Significance levels: *P < 0.05, **P < 0.01, ***P < 0.001.

The participants were required to provide a comprehension rating for each presented word. Figure 2 illustrates the comprehension ratings for all groups, ears, sessions and conditions. Overall, the comprehension rating in both ears of CI users as well as normal hearing controls decreased as the words became more degraded (all *P* < 0.05, Supplementary Tables S2–S4) with one exception: there was no difference between the difficult and very difficult conditions in the CI ears (*z* = 1.91, *P* = 0.22, Supplementary Table S4).

When testing the effects of CI, session, condition and their interactions on CI users’ CI ears and non-CI ears, there were significant main effects for all those factors (all *P* < 0.001, Supplementary Table S5) and significant interactions between CI and session [***χ***^2^(2) = 34.14, *P* < 0.001] as well as between CI and condition [***χ***^2^(3) = 459.96, *P* < 0.001]. These results indicate that the comprehension rating for the non-CI ears improved more over the course of a year and across various levels of degraded words compared to the CI ears.

For the non-CI ears, the comprehension ratings were lower than those of normal hearing controls in all the conditions during the pre-Op session and post-Op 3-mon sessions (all *P* < 0.05, Supplementary Table S6). The session effect in the non-CI ears was significant [***χ***^2^(3) = 393.01, *P* < 0.001], although there were no differences between the pre-op and post-Op 3-month sessions (*z* = −0.17, *P* = 0.99). The condition effect was also significant [***χ***^2^(3) = 209.67, *P* < 0.001] and all the conditions were significantly different from each other (all *P* < 0.001, Supplementary Table S3).

For the CI ears, the comprehension ratings were poorer than those of normal hearing controls in all the conditions across all sessions (all *P* < 0.05, Supplementary Table S7). Nevertheless, the session effect was significant [***χ***^2^(2) = 38.06, *P* < 0.001]. The ratings improved after 6 months of implantation (post-Op 6-month ratings higher than post-Op 3-month with *P* < 0.001; post-Op 6-month ratings higher than post-Op 3-month with *P* < 0.001). The condition effect was also significant [***χ***^2^(3) = 99.03, *P* < 0.001], while there was no significant difference between the difficult and the very difficult conditions (*z* = 1.91, *P* = 0.22). There is no main effect of laterality for both of the non-CI ears and CI ears (Supplementary Tables S8 and S9).

### Decoding accuracies of speech degradation in non-CI ears improved 6 months after implantation

**Figure 3:**
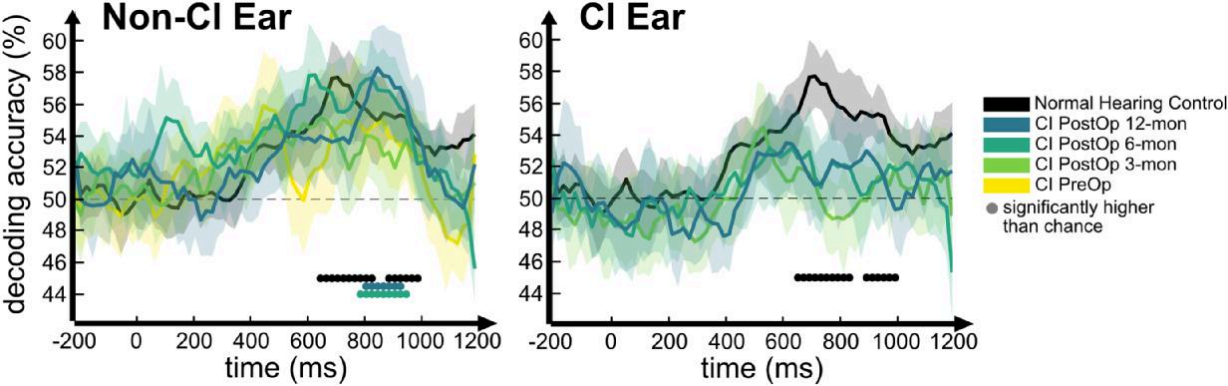
**Average decoding accuracy over time for the non-CI ear (left panel) and the CI ear (right panel) for all conditions.** Results from post-operative non-CI ears along with normal hearing controls showed greater-than-chance decoding accuracy (left panel). On the other hand, CI ears did not show greater-than-chance decoding accuracy in any session (right panel). Rows of dots indicate time points with significantly above-chance decoding accuracy (*N* = 10 for each group; right-tailed sign permutation test, corrected significance level *P* < 0.05).

The average decoding accuracy was significantly above chance (50% chance level for pairwise decoding between every two conditions) during late time points (see Fig. 3). Notably, this was true for normal hearing controls (660–840 and 900–1000 ms) and most post-operative EEG measurement sessions for the non-CI ear (post-Op 6-mon: 800–960 ms; post-Op 12-mon: 820–940 ms). However, for the CI ear, this analysis did not yield above-chance results in any time window. The decoding accuracy between any two conditions in the CI ear could be influenced by the decoding results between the difficult and very difficult conditions, which showed no significant difference in the behavioural results.

### Shared representation among cochlear implant users’ non-cochlear implant ears and normal hearing controls enhanced with comprehension ratings

**Figure 4:**
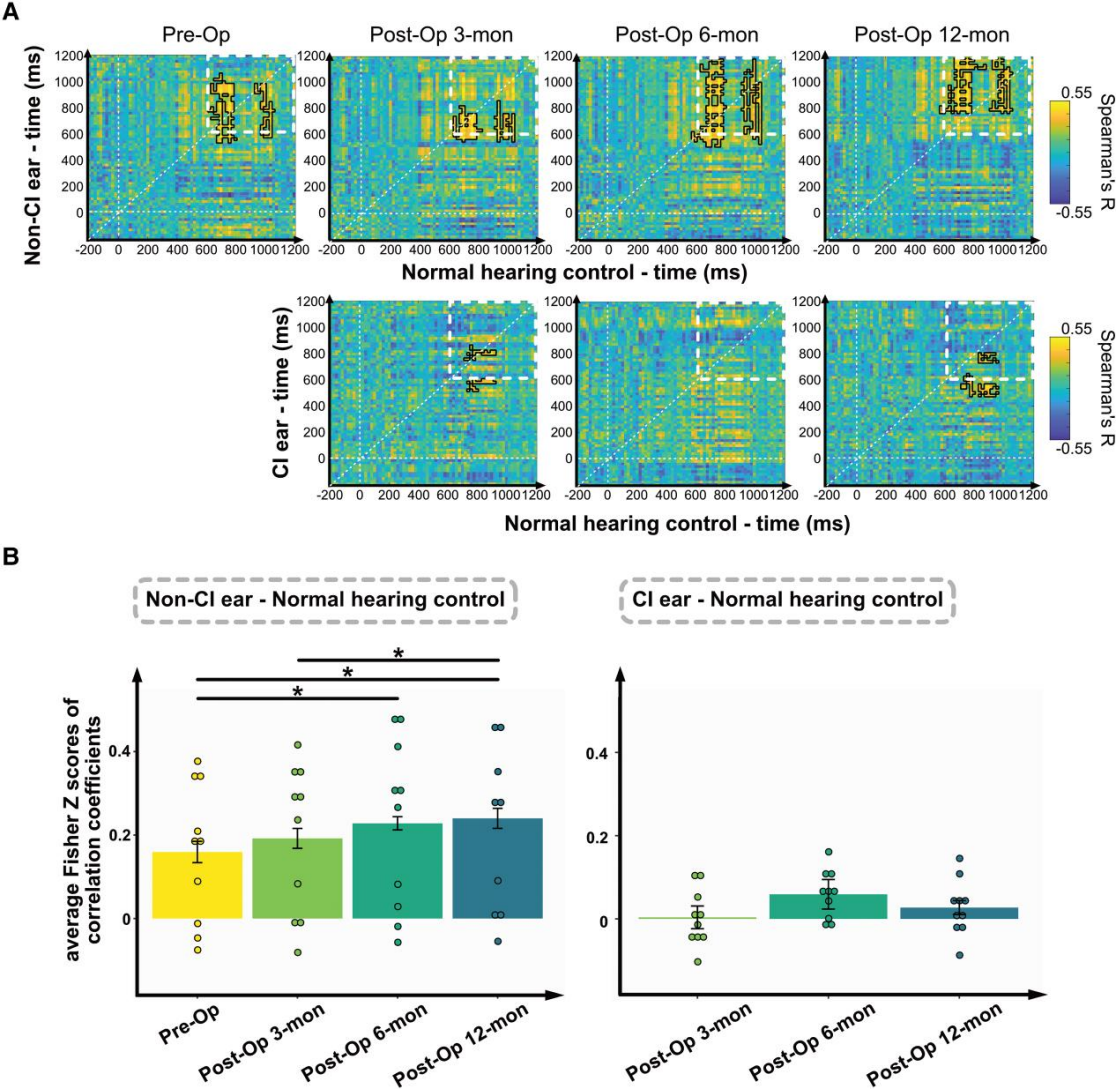
**Quantification of shared degraded speech representation between CI users and normal hearing controls.** (**A**) Average time-generalized Spearman’s R matrices relating CI ears, non-CI ears and normal hearing controls for each session. Significant correlations are highlighted with black lines (*N* = 10 for each group, right-tailed sign permutation tests, cluster-corrected significance level *P* < 0.05). For the result of relating CI users’ CI ear and non-CI ear, see Supplementary Fig. S5. (**B**) Average all of the Fisher *Z*-scores of correlation coefficients within 600 and 1200 ms (white dashed squares in Fig. 4A) of each session and compared them across sessions. The result parallels the findings of decoding accuracy. It shows that the correlation of non-CI ears with normal hearing controls improves at 6- and 12-month post-operation (left panel). On the other hand, CI ears did not show improvement over time (right panel). Bars represent 95% confidence intervals. Significance levels: *P_Bonferroni_ < *α* (see ‘Statistical analysis’; *N* = 10 for each group, permutation paired *t*-tests with Bonferroni correction).

Our results from the subject comprehension rating and the time-resolved classification analysis have shown an improvement in the performance of non-CI ears in processing degraded speech. To further explore how cochlear implantation affects the representation of degraded speech over time, we correlated the RDMs of non-CI ears, CI ears and normal hearing controls. The correlation matrices between the non-CI ears and normal hearing controls in late time windows showed significance above zero in all sessions (Fig. 4A, top row). However, between the CI ear and normal hearing controls, no significant correlations were observed after CI implantation (Fig. 4A, bottom row). Furthermore, to quantify how the correlation may evolve across sessions, we calculated the average of 961 Fisher *Z*-scores of correlation coefficients within the 600–1200 ms time range of the time-generalized Spearman’s R matrices (indicated by white dashed squares in Fig. 4A) for each session. The time window was defined based on the results of the time-resolved classification here and the findings of intelligibility classification from Obleser and Weisz.^44^ For the non-CI ear, the resulting correlations were significantly higher at post-Op 6-mon compared to the pre-Op [*P* < *α* (0.0083), permutation paired *t*-test, Cohen’s *d* = 1.50] and post-Op 12-mon compared to the pre-Op [*P* < *α* (0.0083), *d* = 1.51] and they were higher at post-Op 12-mon than at post-Op 3-mon [*P* < *α* (0.0083), *d* = 0.9] (Fig. 4B). For the CI ear, there was a main effect of session, but no significant results from pairwise comparisons [*F*(2,18) = 5.204, *P* = 0.016, permutation *F*-test] (Fig. 4B).

## Discussion

In this study, we investigated the impact of CIs on neurobehavioral speech processing using RSA to track brain activity patterns related to speech processing over time. We examined neural speech processing in both the non-CI ear and the CI ear, comparing the results to normal hearing control data. This study aimed to reveal the neuroplastic dynamics triggered by CI implantation over multiple time points, including pre-operation and 3, 6 and 12 months after the operation. Our results highlight significant improvements in speech comprehension over a year for cochlear implant users in line with our previously published results from the Oldenburg sentence test conducted with the same participants.^46^ The EEG RSA results mirrored these functional improvements: time-resolved classification analysis of all speech comprehension difficulty levels (i.e. ‘very easy’, ‘easy’, ‘difficult’ and ‘very difficult’) showed above-chance decoding accuracy during late time points (around 600–1000 ms after stimulus onset) for normal hearing controls and 6-month as well as 12-month post-operative EEG measurement sessions for the non-CI ear, but not for the CI ear. These results demonstrate enhanced cortical processing for differentiating various levels of degraded speech presented to the non-CI ears. Furthermore, the quantification of shared degraded speech representation between CI users and normal hearing controls through matrix correlation links the observed improvement in decoding accuracy for the non-CI ear to representational neural patterns becoming more similar to normal hearing individuals. Overall, our results demonstrate that CIs not only provide auditory input to the deaf ear of people with SSD, but crucially, improves speech comprehension outcomes by enhancing the performance of their hearing ear.

### Impact of cochlear implant on the non-cochlear implant ear of stimulus

#### Single-sided deafness individuals

The observed differences in behavioural improvement and neural activity patterns, while not being inter-correlated, with the CI and non-CI ears underscore the importance of considering both ears in the assessment of CI effectiveness and neural processing. In the CI ears, our behavioural results show improvement at 6 and 12 months after the operation, in line with previous studies.^17,19,63^ Importantly, the significant differences compared to the normal hearing controls at the pre-operation and 3 months post-operation as well as the significant improvements observed in speech comprehension over time in the non-CI ears suggest that CIs can lead to functional reorganization of auditory processing in the non-CI ears of SSD individuals. This further emphasizes the potential benefits of CIs for individuals with auditory disorders and highlights the importance of rapid intervention after SSD onset to potentially optimize speech comprehension and other beneficial auditory outcomes, such as pitch perception.^64,65^

In the context of our study on single-sided CI, the significant influence of the non-CI ear on speech comprehension can be interpreted within a Bayesian framework.^66-68^ Since the auditory input from the clinically healthy, non-CI ear continues to dominate over that from the deaf ear, even when aided by a CI, the brain tends to prioritize the information from the non-CI ear. The CI primarily functions as a supplementary source, possibly enhancing the precision of auditory input obtained from the non-CI ear and, consequently, improving overall auditory functioning (here: speech comprehension).

### Electrophysiological signatures of plasticity after cochlear implantation for stimulus

#### Single-sided deafness individuals

Given the novelty of our RSA approach for the SSD-CI EEG data, it is difficult to directly compare our results to previous studies. As ERP measures primarily capture information encoded by activity time-locked to the stimulus, RSA captures information that is variable across trials in a multivariate way, considering interrelations between various features. Therefore, it is difficult to relate these two measures to each other. However, our findings generally align with prior research regarding behavioural improvements and normalization after CI implantation.^29-31^ In these studies, ERP components (e.g. N1 and P2) are related to early processing between 100 and 300 ms, while our data show effects after 600 ms in CI users. These late components, such as the P600, are usually known for being relevant in a higher order of language processing hierarchy (e.g. syntactic) rather than basic acoustic or linguistic processing.^69,70^ In addition, Obleser and Weisz^44^ from which we adapted the study design and the materials for the current study, also showed the maximum effects around 600 ms. Therefore, it is plausible to argue that our results demonstrate the restoration of neural activity in higher levels of speech processing in non-CI ears. Concerning different types of plasticity following CI implantation, our results can only be interpreted as adaptive plasticity since the current experimental design does not enable us to theorize in more depth about the possible involvement of cross-modal (audio–visual) plasticity.^33,34,37^

In our results of the time-generalized similarity analysis, we observed an increase in correlation up to 6 and 12 months post-CI implantation. Kral and Sharma^36^ emphasize that factors such as the duration and onset of auditory deprivation can affect the time course of neuroplastic changes. However, longitudinal EEG data on adult CI-SSD cases are so scarce that it is challenging to draw general conclusions about these critical time windows. In our sample, 9 out of 10 participants received their CI within 2 years after the onset of SSD, which can be considered an early intervention where neuroplasticity should be plausible. We therefore conclude that the results of our study reflect such neuroplastic processes, although we cannot completely rule out the influence of individual differences in our small sample.

### Application of representational similarity analysis on tracking neuroplasticity

Our findings underscore the utility of RSA as a powerful tool for investigating neuroplastic changes in the brain following CI implantation. This method enables more specific and detailed tracking of neuroplasticity effects, providing valuable insights into the neural mechanisms underlying improvements in speech comprehension and auditory functioning in general.^71^ Consequently, RSA may prove beneficial in other clinical and longitudinal use cases, especially with small sample sizes, for studying auditory disorders and interventions.^39^ Our applied methodology can thus potentially inform the development of more effective diagnosis, rehabilitation and intervention strategies by using healthy controls (between subjects) or healthy state data (within subjects) as reference decoding data across multiple time points.

### Limitations and future directions

The study encountered several limitations, which we address below to support the validity and reliability of our findings. First, the small sample size of 10 participants per group (i.e. CI users and normal hearing controls), although well-matched with normal hearing controls and comprehensively documented, is not uncommon in CI research, especially in longitudinal studies. This small sample size and the less optimal experiment design limit us to correlate behavioural data with neural data on a per-subject basis. Future research should consider larger samples and better experimental designs (e.g. more distinction in comprehensibility across conditions and more trials per condition) to better relate individual neural observations to behavioural performance. Second, dealing with noisy CI EEG data presented a significant challenge. However, we employed ICA cleaning methods with expert care to ensure data quality.^53,72-74^ Third, no EEG changes for the CI ear were detected, which could be due to the exclusive use of spectrally degraded stimuli. Future research should consider including non-degraded words/speech, which are also able to induce larger and more specific neural responses as well as possibly better align with the behavioural results. Fourth, while we observed improvement in the Oldenburg sentence test and sound localization test,^46^ as well as lack of a condition and session interaction in the degraded word rating (i.e. the investigated variable of this study), we cannot rule out that the observed improvement in the non-CI ear may have arisen from a learning effect since corresponding data across sessions from a control group was not available. Future studies should consider better control for learning effects, for example, by including a control group with SSD before implantation to assess neuroplasticity unrelated to CIs and their auditory stimulation, or by measuring normal hearing controls over time. However, the increasing neural similarity in speech processing between the non-CI ear of CI users and healthy controls cannot be solely attributed to a learning effect from stimulus exposure. Lastly, the heterogeneity among CI individuals, such as CI efficacy and tolerability, wearing time and so on, may have impacted the results, particularly given the small sample size and considerable age range. Despite these unavoidable challenges, the study yielded plausible and consistent results across analyses.

## Conclusion

In the present study, we demonstrated the normalization of neural representation patterns in response to speech stimuli over time after CI implantation in adults with SSD, particularly in the non-CI ear, using RSA. The gradual improvement in behavioural speech comprehension performance was reflected in enhancement of shared degraded speech representation between CI users and normal hearing controls. Taken together, our findings confirm the positive effects on speech comprehension after CI implantation and advocate for the application of RSA in future clinical longitudinal studies. Ultimately, these advancements could also encourage CI users to use their devices more frequently, maximizing the benefits they receive from their implants.

## Supplementary Material

fcaf001_Supplementary_Data

## Data Availability

The data and code necessary for generating the figures and computing statistics will be shared in the corresponding author’s gitlab repository: https://gitlab.com/yapingchen/longitudinal-ssd-ci. Access to raw data will be made available upon reasonable request to the corresponding author.
