# Supplementary material for: Cochlear implantation in adults with acquired single-sided deafness improves cortical processing and comprehension of speech presented to the non-implanted ears: a longitudinal EEG study
Source: Brain Commun. 2025 Jan 3;7(1):fcaf001. doi: 10.1093/braincomms/fcaf001 (PMC11733687; doi:10.1093/braincomms/fcaf001)
Supplement: fcaf001_Supplementary_Data [file fcaf001_supplementary_data.pdf]

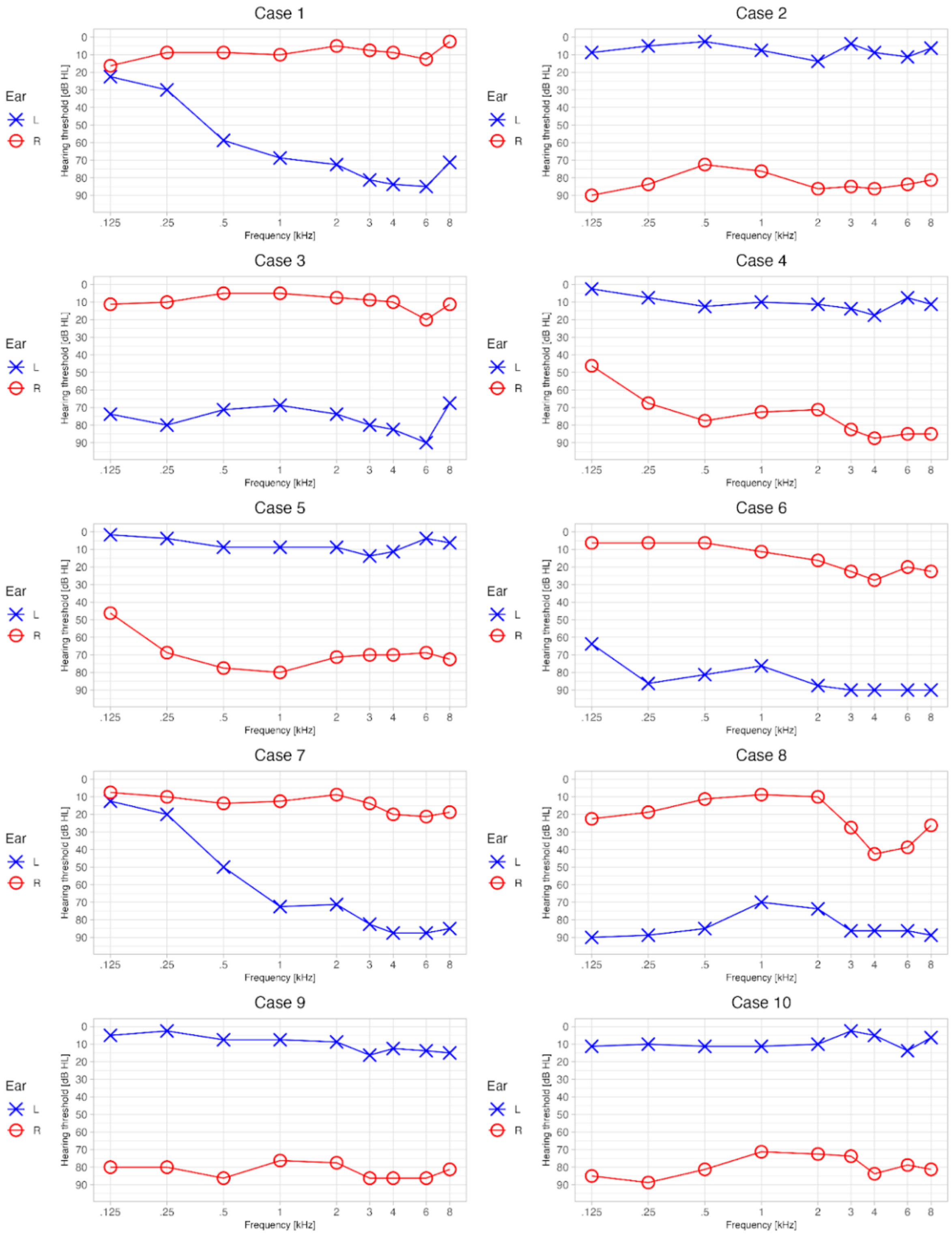

Supplementary Figure 1 Pure-tone audiograms of CI Cases before CI implantation. Hearing is preserved and normal in the healthy ear except some mild hearing loss at high frequencies in cases 6 and 8.

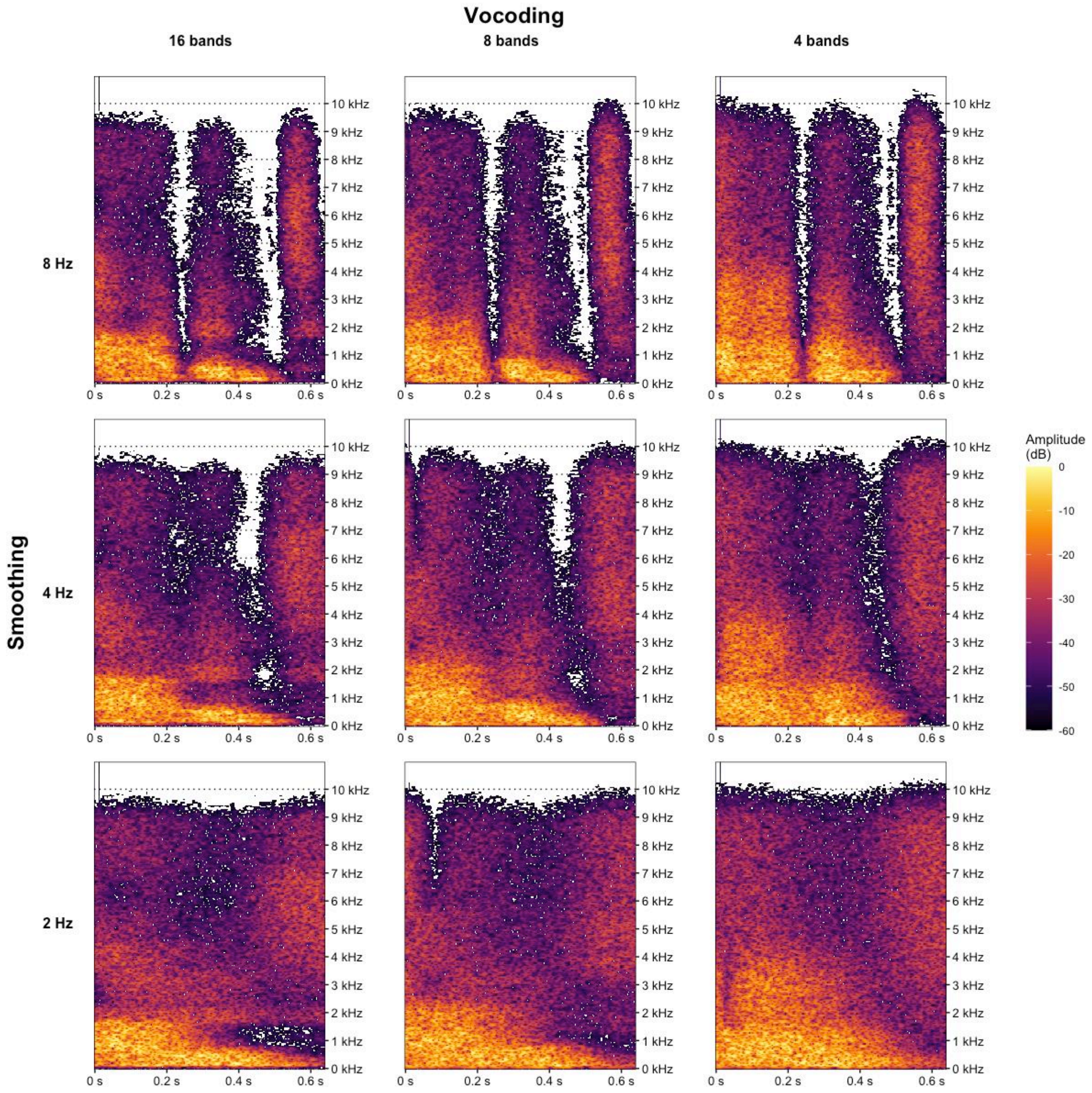

Supplementary Figure 2 Spectrograms of stimulus material. Spectral and temporal features of an exemplary original sound (German word "Abend") degrade from left to right and from top to bottom, following x and y axis, respectively. Stimuli are normalized to full scale. Note that sounds are represented up to 11.025 kHz due to the original audio sampling rate of 22.05 kHz.

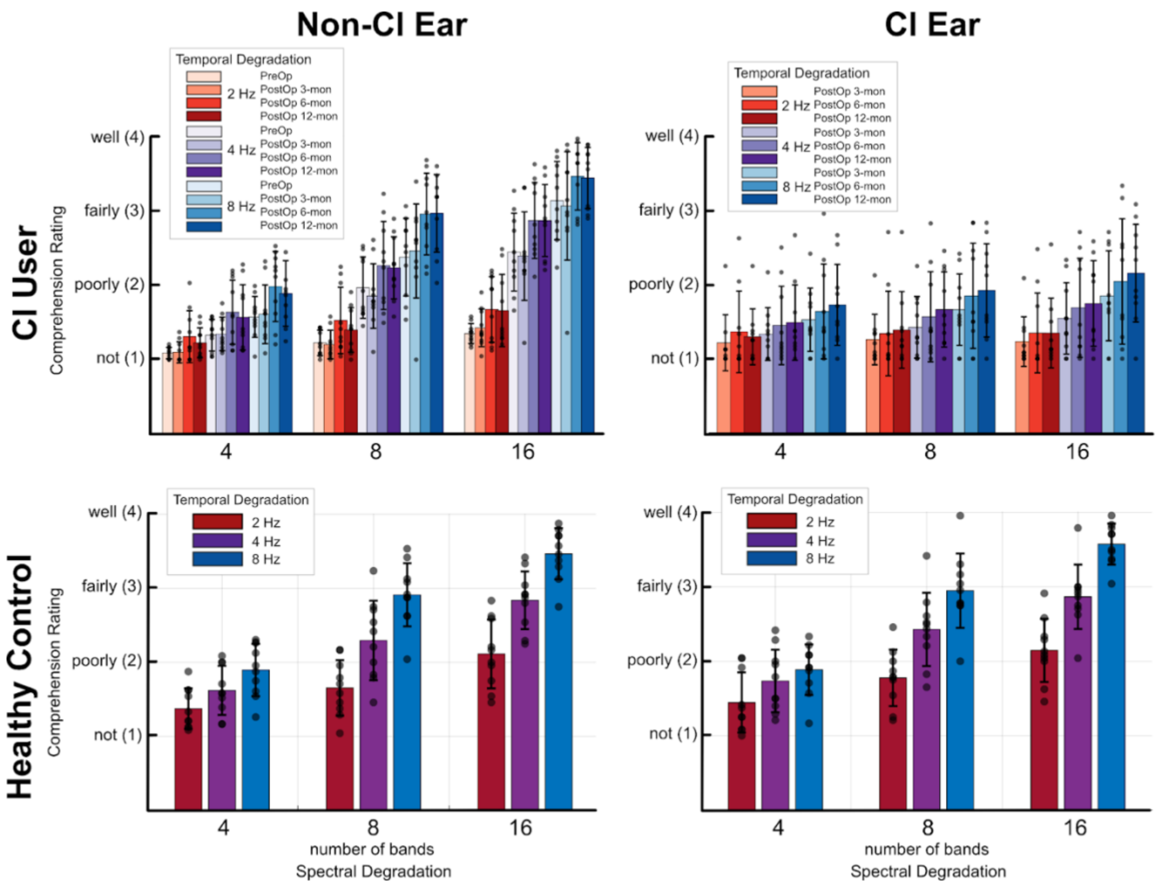

Supplementary Figure 3 Behavioral results in the original 9-condition design.

The results here align with those that we aggregated into four conditions (see Results). There is a significant interaction between the effect of CI and session (N=10 for each group, cumulative link mixed models), indicating that comprehension ratings for the non-CI ears improved more over the course of a year compared to the CI ears (Supplementary Table 1.1). For the non-CI ears, comprehension ratings were lower than those of normal hearing controls at the Pre-Op and Post-Op 3-month sessions but not at the Post-Op 6-month and Post-Op 12-month sessions (Supplementary Table 1.2).

Supplementary Table 1.1 A cumulative link mixed model for examining the effect of CI, session, and condition as well as the interaction between those main effects within CI participants in the 9-condition design:

rating ~ CI * session * condition + (1|item) + (1|subject).

CI ear vs. non-CI ear					
**Regression weights**	**Estimate**	**Std. Err.**	**z value**	***P***	
CI ear	1.005	0.311	3.226	0.001	**
post-op 6-mon	1.400	0.298	4.699	<0.001	***
post-op 12-mon	0.993	0.307	3.233	0.001	**
condition 4-band 4-Hz	1.569	0.353	4.442	<0.001	***
condition 4-band 8-Hz	2.448	0.344	7.108	<0.001	***
condition 8-band 2-Hz	0.854	0.368	2.318	0.020	*
condition 8-band 4-Hz	3.114	0.342	9.099	<0.001	***
condition 8-band 8-Hz	4.393	0.341	12.867	<0.001	***
condition 16-band 2-Hz	1.899	0.351	5.406	<0.001	***
condition 16-band 4-Hz	4.258	0.341	12.492	<0.001	***
condition 16-band 8-Hz	5.677	0.344	16.500	<0.001	***
CI ear:post-op 6-mon	-0.861	0.380	-2.265	0.024	*
CI ear:post-op 12-mon	-0.794	0.392	-2.024	0.043	*
CI ear:condition 4-band 4-Hz	-1.012	0.380	-2.664	0.008	**
CI ear:condition 4-band 8-Hz	-1.278	0.366	-3.493	<0.001	***
CI ear:condition 8-band 2-Hz	-0.578	0.398	-1.451	0.147	
CI ear:condition 8-band 4-Hz	-2.284	0.367	-6.227	<0.001	***
CI ear:condition 8-band 8-Hz	-2.851	0.359	-7.933	<0.001	***
CI ear:condition 16-band 2-Hz	-1.766	0.386	-4.574	<0.001	***
CI ear:condition 16-band 4-Hz	-2.993	0.361	-8.285	<0.001	***
CI ear:condition 16-band 8-Hz	-3.655	0.360	-10.152	<0.001	***
post-op 6-mon:condition 4-band 4-Hz	-0.511	0.360	-1.419	0.156	
post-op 12-mon:condition 4-band 4-Hz	-0.288	0.369	-0.781	0.435	
post-op 6-mon:condition 4-band 8-Hz	-0.569	0.349	-1.633	0.102	
post-op 12-mon:condition 4-band 8-Hz	-0.361	0.358	-1.008	0.314	
post-op 6-mon:condition 8-band 2-Hz	-0.109	0.377	-0.289	0.773	

post-op 12-mon:condition 8-band 2-Hz	-0.109	0.388	-0.280	0.779	
post-op 6-mon:condition 8-band 4-Hz	-0.659	0.346	-1.903	0.057	.
post-op 12-mon:condition 8-band 4-Hz	-0.253	0.355	-0.712	0.477	
post-op 6-mon:condition 8-band 8-Hz	-0.469	0.345	-1.359	0.174	
post-op 12-mon:condition 8-band 8-Hz	0.019	0.354	0.054	0.957	
post-op 6-mon:condition 16-band 2-Hz	-0.735	0.358	-2.054	0.040	*
post-op 12-mon:condition 16-band 2-Hz	-0.389	0.366	-1.064	0.287	
post-op 6-mon:condition 16-band 4-Hz	-0.570	0.344	-1.656	0.098	.
post-op 12-mon:condition 16-band 4-Hz	-0.090	0.352	-0.255	0.799	
post-op 6-mon:condition 16-band 8-Hz	-0.497	0.352	-1.410	0.158	
post-op 12-mon:condition 16-band 8-Hz	-0.035	0.362	-0.098	0.922	
CI ear:post-op 6-mon:condition 4-band 4-Hz	0.308	0.479	0.643	0.520	
CI ear:post-op 12-mon:condition 4-band 4-Hz	0.474	0.489	0.968	0.333	
CI ear:post-op 6-mon:condition 4-band 8-Hz	0.283	0.463	0.611	0.541	
CI ear:post-op 12-mon:condition 4-band 8-Hz	0.589	0.473	1.245	0.213	
CI ear:post-op 6-mon:condition 8-band 2-Hz	-0.196	0.499	-0.392	0.695	
CI ear:post-op 12-mon:condition 8-band 2-Hz	0.161	0.511	0.315	0.753	
CI ear:post-op 6-mon:condition 8-band 4-Hz	0.478	0.465	1.028	0.304	
CI ear:post-op 12-mon:condition 8-band 4-Hz	0.642	0.474	1.355	0.175	
CI ear:post-op 6-mon:condition 8-band 8-Hz	0.260	0.456	0.570	0.568	
CI ear:post-op 12-mon:condition 8-band 8-Hz	0.261	0.467	0.559	0.576	
CI ear:post-op 6-mon:condition 16-band 2-Hz	0.614	0.487	1.261	0.207	
CI ear:post-op 12-mon:condition 16-band 2-Hz	0.446	0.498	0.895	0.371	
CI ear:post-op 6-mon:condition 16-band 4-Hz	0.212	0.459	0.461	0.645	
CI ear:post-op 12-mon:condition 16-band 4-Hz	0.217	0.469	0.463	0.644	
CI ear:post-op 6-mon:condition 16-band 8-Hz	0.226	0.460	0.491	0.623	
CI ear:post-op 12-mon:condition 16-band 8-Hz	0.410	0.471	0.870	0.384	

reference category: non-CI ear, condition very difficult, post-op 3-mon

**Analysis of Deviance**	**LR Chisq**	**Df**	***P***	
CI	1218.720	1	<0.001	***
session	232.710	2	<0.001	***
condition	295.540	8	<0.001	***

CI:session	41.840	2	<0.001	***
CI:condition	771.440	8	<0.001	***
session:condition	15.900	16	0.460	
CI:session:condition	7.360	16	0.966	

Post-op, post-operation; CI, cochlear implant.

Supplementary Table 1.2 A cumulative link mixed model for examining the effect of CI and condition between normal hearing controls' and the non-CI ears' subjective comprehension ratings in the 9-condition design:

rating ~ group*condition + (1|item) + (1|subject).

non-CI ear vs. normal hearing control					
**Pre-Op**					
**Regression weights**	**Estimate**	**Std. Err.**	**z value**	***P***	
group NHC	2.037	0.470	4.336	<0.001	***
condition 4-band 4-Hz	1.500	0.418	3.589	<0.001	***
condition 4-band 8-Hz	2.605	0.409	6.366	<0.001	***
condition 8-band 2-Hz	1.126	0.426	2.647	0.008	**
condition 8-band 4-Hz	3.750	0.407	9.205	<0.001	***
condition 8-band 8-Hz	4.828	0.408	11.831	<0.001	***
condition 16-band 2-Hz	1.788	0.415	4.310	<0.001	***
condition 16-band 4-Hz	4.985	0.407	12.248	<0.001	***
condition 16-band 8-Hz	6.764	0.413	16.368	<0.001	***
group NHC:condition 4-band 4-Hz	-0.542	0.328	-1.653	0.098	.
group NHC:condition 4-band 8-Hz	-0.484	0.316	-1.533	0.125	
group NHC:condition 8-band 2-Hz	-0.235	0.338	-0.697	0.486	
group NHC:condition 8-band 4-Hz	-1.054	0.313	-3.371	<0.001	***
group NHC:condition 8-band 8-Hz	-0.941	0.312	-3.013	0.003	**
group NHC:condition 16-band 2-Hz	-0.302	0.325	-0.931	0.352	
group NHC:condition 16-band 4-Hz	-0.905	0.310	-2.915	0.004	**
group NHC:condition 16-band 8-Hz	-0.871	0.321	-2.712	0.007	**
reference category: group non-CI ear, condition 4-band 2-Hz					
**Analysis of Deviance**		**LR Chisq**	**Df**	***P***	
group		9.810	1	0.002	**
condition		297.060	8	<0.001	***
group:condition		29.158	8	<0.001	***
**Post-Op 3-mon**					
**Regression weights**	**Estimate**	**Std. Err.**	**z value**	***P***	
group NHC	2.526	0.626	4.035	<0.001	***
condition 4-band 4-Hz	1.841	0.435	4.233	<0.001	***

condition 4-band 8-Hz	2.919	0.427	6.845	<0.001	***
condition 8-band 2-Hz	1.047	0.448	2.338	0.019	*
condition 8-band 4-Hz	3.763	0.425	8.862	<0.001	***
condition 8-band 8-Hz	5.490	0.426	12.889	<0.001	***
condition 16-band 2-Hz	2.244	0.432	5.190	<0.001	***
condition 16-band 4-Hz	5.282	0.425	12.431	<0.001	***
condition 16-band 8-Hz	7.095	0.430	16.487	<0.001	***
group NHC:condition 4-band 4-Hz	-0.885	0.346	-2.562	0.010	*
group NHC:condition 4-band 8-Hz	-0.788	0.334	-2.362	0.018	*
group NHC:condition 8-band 2-Hz	-0.160	0.362	-0.443	0.658	
group NHC:condition 8-band 4-Hz	-1.038	0.331	-3.136	0.002	**
group NHC:condition 8-band 8-Hz	-1.560	0.330	-4.727	<0.001	***
group NHC:condition 16-band 2-Hz	-0.757	0.343	-2.211	0.027	*
group NHC:condition 16-band 4-Hz	-1.157	0.329	-3.515	<0.001	***
group NHC:condition 16-band 8-Hz	-1.110	0.338	-3.280	0.001	**

reference category: group non-CI ear, condition 4-band 2-Hz

**Analysis of Deviance**	**LR Chisq**	**Df**	***P***	
group	7.001	1	0.008	**
condition	299.790	8	<0.001	***
group:condition	44.822	8	<0.001	***

Post-Op 6-mon

**Regression weights**	**Estimate**	**Std. Err.**	**z value**	***P***	
group NHC	0.601	0.479	1.255	0.210	
condition 4-band 4-Hz	1.235	0.343	3.599	<0.001	***
condition 4-band 8-Hz	2.280	0.341	6.696	<0.001	***
condition 8-band 2-Hz	0.875	0.346	2.533	0.011	*
condition 8-band 4-Hz	3.003	0.341	8.820	<0.001	***
condition 8-band 8-Hz	4.794	0.345	13.906	<0.001	***
condition 16-band 2-Hz	1.362	0.343	3.968	<0.001	***
condition 16-band 4-Hz	4.533	0.343	13.214	<0.001	***
condition 16-band 8-Hz	6.325	0.356	17.742	<0.001	***
group NHC:condition 4-band 4-Hz	-0.290	0.253	-1.145	0.252	
group NHC:condition 4-band 8-Hz	-0.191	0.247	-0.772	0.440	
group NHC:condition 8-band 2-Hz	-0.010	0.257	-0.038	0.969	

group NHC:condition 8-band 4-Hz	-0.345	0.247	-1.399	0.162	
group NHC:condition 8-band 8-Hz	-0.959	0.249	-3.850	<0.001	***
group NHC:condition 16-band 2-Hz	0.090	0.254	0.354	0.724	
group NHC:condition 16-band 4-Hz	-0.508	0.247	-2.054	0.040	*
group NHC:condition 16-band 8-Hz	-0.505	0.267	-1.888	0.059	.

reference category: group non-CI ear, condition 4-band 2-Hz

**Analysis of Deviance**	**LR Chisq**	**Df**	***P***	
group	0.400	1	0.526	
condition	316.800	8	<0.001	***
group:condition	31.640	8	<0.001	***

Post-Op 12-mon

**Regression weights**	**Estimate**	**Std. Err.**	**z value**	***P***	
group NHC	1.037	0.479	2.164	0.030	*
condition 4-band 4-Hz	1.434	0.366	3.912	<0.001	***
condition 4-band 8-Hz	2.456	0.363	6.763	<0.001	***
condition 8-band 2-Hz	0.847	0.372	2.279	0.023	*
condition 8-band 4-Hz	3.452	0.365	9.462	<0.001	***
condition 8-band 8-Hz	5.293	0.368	14.385	<0.001	***
condition 16-band 2-Hz	1.758	0.365	4.816	<0.001	***
condition 16-band 4-Hz	4.982	0.365	13.651	<0.001	***
condition 16-band 8-Hz	6.824	0.380	17.951	<0.001	***
group NHC:condition 4-band 4-Hz	-0.493	0.268	-1.840	0.066	.
group NHC:condition 4-band 8-Hz	-0.375	0.262	-1.433	0.152	
group NHC:condition 8-band 2-Hz	0.032	0.276	0.115	0.909	
group NHC:condition 8-band 4-Hz	-0.809	0.263	-3.075	0.002	**
group NHC:condition 8-band 8-Hz	-1.482	0.264	-5.615	<0.001	***
group NHC:condition 16-band 2-Hz	-0.297	0.266	-1.116	0.264	
group NHC:condition 16-band 4-Hz	-0.974	0.260	-3.746	<0.001	***
group NHC:condition 16-band 8-Hz	-0.979	0.282	-3.467	<0.001	***

reference category: group non-CI ear, condition 4-band 2-Hz

**Analysis of Deviance**	**LR Chisq**	**Df**	***P***	
group	0.850	1	0.357	
condition	308.741	8	<0.001	***
group:condition	67.631	8	<0.001	***

Pre-op, post-operation; post-op, post-operation; CI, cochlear implant; NHC, normal hearing control.

Supplementary Text 1 Event-related potential analysis and result.

We conducted the ERP analysis primarily as a sanity check. Our ERP results are not directly comparable to the literature, which typically uses clear clicks or words, each repeated more than 100 times. The EEG data preprocessing and CI artifact suppression were the same as those used for representational similarity analysis (RSA). The preprocessed data were segmented into epochs from -500 to 1000 ms relative to the word onset, with baseline correction applied by subtracting the mean from the 100 ms prestimulus period. ERPs were obtained by averaging data from 216 trials across all conditions. We calculated the global field power (GFP), representing the variance across electrodes, for all electrodes. All analyses were performed using Matlab and the Fieldtrip toolbox. Due to the acoustical degradation of the words, the latency of the first negative ERP peaks (~200 ms) is generally more delayed compared to the typical N1 components (~100 ms)(Supplementary Figure 3). Moreover, there is no late effect (around 600 ms) observed here that we noted in the RSA results and Obleser et al.[1]

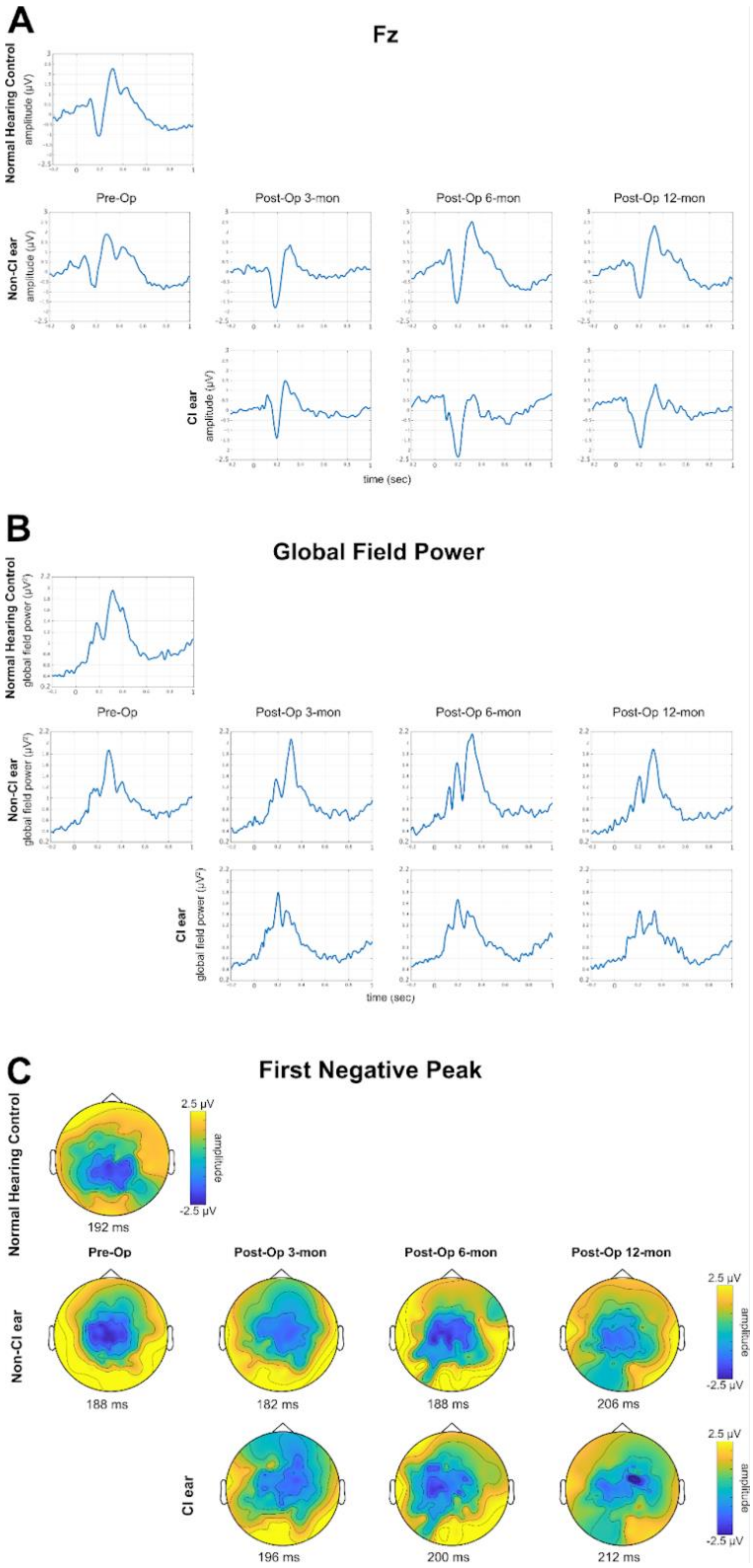

Supplementary Figure 4 Event-related potential results. (A) The cortical response at the Fz electrode was averaged for each group and session by pooling all trials (N=10 for each group). (B) The global field power was averaged for each group and session by pooling all trials. (C) Topographic distribution of mean average-referenced EEG activity at the first negative peak latency.

Supplementary Table 2 A cumulative link mixed model for examining the effect of condition in normal hearing controls:

rating ~ condition + (1|item) + (1|subject).

Normal Hearing Control					
**Regression weights**	**Estimate**	**Std. Err.**	**z value**	***P***	
condition difficult	0.782	0.228	3.434	<0.001	***
condition easy	2.003	0.229	8.766	<0.001	***
condition very easy	3.631	0.234	15.543	<0.001	***
reference category: condition very difficult					
**Comparisons of Condition**	**Estimate**	**Std. Err.**	**z value**	***P***	
very difficult - difficult	-0.782	0.228	-3.434	0.003	**
very difficult - easy	-2.003	0.228	-8.766	<0.001	***
very difficult - very easy	-3.631	0.234	-15.543	<0.001	***
difficult - easy	-1.221	0.226	-5.402	<0.001	***
difficult - very easy	-2.848	0.230	-12.39	<0.001	***
easy - very easy	-1.628	0.226	-7.216	<0.001	***

Supplementary Table 3 A cumulative link mixed model for examining the effect of condition and session in non-CI ears:

rating ~ condition * session + (1|item) + (1|subject).

Non-CI ear					
**Regression weights**	**Estimate**	**Std. Err.**	**z value**	***P***	
post-op 3-mon	-0.112	0.209	-0.534	0.593	
post-op 6-mon	1.334	0.179	7.468	<0.001	***
post-op 12-mon	0.880	0.184	4.779	<0.001	***
condition difficult	1.036	0.276	3.758	<0.001	***
condition easy	2.441	0.269	9.067	<0.001	***
condition very easy	4.000	0.269	14.881	<0.001	***
post-op 3-mon:condition difficult	0.309	0.261	1.184	0.236	
post-op 6-mon:condition difficult	-0.248	0.230	-1.075	0.282	
post-op 12-mon:condition difficult	0.047	0.235	0.200	0.842	
post-op 3-mon:condition easy	0.040	0.247	0.163	0.871	
post-op 6-mon:condition easy	-0.473	0.219	-2.154	0.031	*
post-op 12-mon:condition easy	-0.149	0.225	-0.661	0.508	
post-op 3-mon:condition very easy	0.150	0.243	0.619	0.536	
post-op 6-mon:condition very easy	-0.269	0.216	-1.243	0.214	
post-op 12-mon:condition very easy	0.243	0.222	1.097	0.273	
reference category: pre-op, condition very difficult					
**Analysis of Deviance**		**LR Chisq**	**Df**	***P***	
session		393.010	3	<0.001	***
condition		209.670	3	<0.001	***
session:condition		15.310	9	0.083	
**Comparisons of Session**	**Estimate**	**Std. Err.**	**z value**	***P***	
pre-op - post-op 3-mon	-0.013	0.079	-0.165	0.998	
pre-op - post-op 6-mon	-1.087	0.073	-14.827	<0.001	***
pre-op - post-op 12-mon	-0.915	0.074	-12.317	<0.001	***
post-op 3-mon - post-op 6-mon	-1.074	0.075	-14.416	<0.001	***
post-op 3-mon - post-op 12-mon	-0.902	0.076	-11.950	<0.001	***
post-op 6-mon - post-op 12-mon	0.172	0.068	2.540	0.054	.
**Comparisons of Condition**	**Estimate**	**Std. Err.**	**z value**	***P***	
very difficult - difficult	-1.060	0.223	-4.774	<0.001	***

very difficult - easy	-2.300	0.222	-10.350	<0.001	***
very difficult - very easy	-4.030	0.224	-18.004	<0.001	***
difficult - easy	-1.230	0.216	-5.697	<0.001	***
difficult - very easy	-2.970	0.218	-13.627	<0.001	***
easy - very easy	-1.740	0.214	-8.093	<0.001	***

Pre-op, post-operation; post-op, post-operation; CI, cochlear implant.

Supplementary Table 4 A cumulative link mixed model for examining the effect of condition and session in CI ears:

rating ~ condition * session + (1|item) + (1|subject).

CI ear					
**Regression weights**	**Estimate**	**Std. Err.**	**z value**	***P***	
post-op 6-mon	0.403	0.170	2.375	0.018	*
post-op 12-mon	0.256	0.173	1.479	0.139	
condition difficult	0.244	0.207	1.179	0.239	
condition easy	0.978	0.199	4.911	<0.001	***
condition very easy	1.391	0.196	7.089	<0.001	***
post-op 6-mon:condition difficult	0.010	0.234	0.043	0.966	
post-op 12-mon:condition difficult	0.109	0.237	0.461	0.645	
post-op 6-mon:condition easy	-0.068	0.223	-0.303	0.762	
post-op 12-mon:condition easy	0.331	0.225	1.473	0.141	
post-op 6-mon:condition very easy	-0.097	0.219	-0.442	0.658	
post-op 12-mon:condition very easy	0.242	0.222	1.090	0.276	
reference category: post-op 3-mon, condition very difficult					
**Analysis of Deviance**		**LR Chisq**	**Df**	***P***	
session		38.057	2	<0.001	***
condition		99.033	3	<0.001	***
session:condition		5.112	6	0.530	
**Comparisons of Session**	**Estimate**	**Std. Err.**	**z value**	***P***	
post-op 3-mon - post-op 6-mon	-0.364	0.077	-4.726	<0.001	***
post-op 3-mon - post-op 12-mon	-0.426	0.078	-5.464	<0.001	***
post-op 6-mon - post-op 12-mon	-0.062	0.076	-0.820	0.691	
**Comparisons of Condition**	**Estimate**	**Std. Err.**	**z value**	***P***	
very difficult - difficult	-0.284	0.149	-1.908	0.224	
very difficult - easy	-1.066	0.146	-7.274	<0.001	***
very difficult - very easy	-1.440	0.146	-9.850	<0.001	***
difficult - easy	-0.782	0.145	-5.409	<0.001	***
difficult - very easy	-1.156	0.144	-8.022	<0.001	***
easy - very easy	-0.374	0.140	-2.664	0.039	*

Post-Op, post operation; CI, cochlear implant.

Supplementary Table 5 A cumulative link mixed model for examining the effect of CI, session, and condition as well as the interaction between those main effects within CI participants:

rating ~ CI + session + condition + CI:session + CI:condition + (1|item) + (1|subject).

CI ear vs. non-CI ear					
**Regression weights**	**Estimate**	**Std. Err.**	**z value**	***P***	
CI ear	0.409	0.115	3.575	<0.001	***
condition difficult	0.950	0.163	5.825	<0.001	***
condition easy	2.033	0.162	12.546	<0.001	***
condition very easy	3.716	0.164	22.657	<0.001	***
post-op 6-mon	0.901	0.069	12.980	<0.001	***
post-op 12-mon	0.789	0.070	11.301	<0.001	***
CI ear:post-op 6-mon	-0.578	0.101	-5.741	<0.001	***
CI ear:post-op 12-mon	-0.406	0.101	-4.015	<0.001	***
CI ear:condition difficult	-0.697	0.126	-5.523	<0.001	***
CI ear:condition easy	-1.089	0.121	-8.961	<0.001	***
CI ear:condition very easy	-2.420	0.122	-19.809	<0.001	***
reference category: non-CI ear, condition very difficult, post-op 3-mon					
**Analysis of Deviance**		**LR Chisq**	**Df**	***P***	
CI		755.89	1	<0.001	***
session		202.29	3	<0.001	***
condition		196.46	2	<0.001	***
CI:session		34.14	2	<0.001	***
CI:condition		459.96	3	<0.001	***

Post-Op, post operation; CI, cochlear implant.

Supplementary Table 6 A cumulative link mixed model for examining the effect of CI and condition between normal hearing controls' and the non-CI ears' subjective comprehension ratings:

rating ~ condition * group + (1|item) + (1|subject).

non-CI ear vs. normal hearing control					
**Pre-Op**					
**Regression weights**	**Estimate**	**Std. Err.**	**z value**	***P***	
condition difficult	1.087	0.295	3.681	<0.001	***
condition easy	2.655	0.290	9.149	<0.001	***
condition very easy	4.401	0.294	14.997	<0.001	***
group NHC	1.748	0.431	4.058	<0.001	***
condition difficult:group NHC	-0.211	0.233	-0.908	0.364	
condition easy:group NHC	-0.592	0.222	-2.664	0.008	**
condition very easy:group NHC	-0.626	0.222	-2.823	0.005	**
reference category: condition very difficult, group non-CI ear					
**Comparisons of Group for each Condition**	**Estimate**	**Std. Err.**	**z value**	***P***	
Condition = very difficult (non-CI - NHC)	-1.750	0.431	-4.058	<0.001	***
Condition = difficult (non-CI - NHC)	-1.540	0.418	-3.672	<0.001	***
Condition = easy (non-CI - NHC)	-1.160	0.412	-2.805	0.005	**
Condition = very easy (non-CI - NHC)	-1.120	0.411	-2.729	0.006	**
**Post-Op 3-mon**					
**Regression weights**	**Estimate**	**Std. Err.**	**z value**	***P***	
condition difficult	1.497	0.307	4.878	<0.001	***
condition easy	2.866	0.302	9.503	<0.001	***
condition very easy	4.889	0.306	15.964	<0.001	***
group NHC	2.224	0.574	3.877	<0.001	***
condition difficult:group NHC	-0.632	0.244	-2.593	0.010	**
condition easy:group NHC	-0.779	0.234	-3.334	<0.001	***
condition very easy:group NHC	-1.053	0.233	-4.514	<0.001	***
reference category: condition very difficult, group non-CI ear					
**Comparisons of Group for each Condition**	**Estimate**	**Std. Err.**	**z value**	***P***	
Condition = very difficult (non-CI - NHC)	-2.220	0.574	-3.877	<.0001	***
Condition = difficult (non-CI - NHC)	-1.590	0.561	-2.840	0.005	**
Condition = easy (non-CI - NHC)	-1.450	0.556	-2.601	0.009	**

Condition = very easy (non-CI - NHC)	-1.170	0.554	-2.114	0.035	*

Post-Op 6-mon

Regression weights	Estimate	Std. Err.	z value	*P*	
condition difficult	0.887	0.244	3.639	<0.001	***
condition easy	2.252	0.243	9.267	<0.001	***
condition very easy	4.287	0.250	17.122	<0.001	***
group NHC	0.462	0.487	0.949	0.343	
condition difficult:group NHC	-0.046	0.200	-0.229	0.819	
condition easy:group NHC	-0.247	0.194	-1.273	0.203	
condition very easy:group NHC	-0.608	0.196	-3.096	0.002	**
reference category: condition very difficult, group non-CI ear					

Comparisons of Group for each Condition	Estimate	Std. Err.	z value	*P*	
Condition = very difficult (non-CI - NHC)	-0.462	0.487	-0.949	0.343	
Condition = difficult (non-CI - NHC)	-0.416	0.483	-0.861	0.389	
Condition = easy (non-CI - NHC)	-0.214	0.480	-0.446	0.655	
Condition = very easy (non-CI - NHC)	0.146	0.480	0.304	0.761	

Post-Op 12-mon

Regression weights	Estimate	Std. Err.	z value	*P*	
condition difficult	1.190	0.256	4.648	<0.001	***
condition easy	2.535	0.256	9.901	<0.001	***
condition very easy	4.699	0.263	17.881	<0.001	***
group NHC	0.881	0.464	1.898	0.058	
condition difficult:group NHC	-0.349	0.205	-1.699	0.089	
condition easy:group NHC	-0.556	0.201	-2.768	0.006	**
condition very easy:group NHC	-1.077	0.202	-5.332	<0.001	***
reference category: condition very difficult, group non-CI ear					

Comparisons of Group for each Condition	Estimate	Std. Err.	z value	*P*	
Condition = very difficult (non-CI - NHC)	-0.881	0.464	-1.898	0.058	.
Condition = difficult (non-CI - NHC)	-0.531	0.458	-1.161	0.245	
Condition = easy (non-CI - NHC)	-0.324	0.455	-0.713	0.476	
Condition = very easy (non-CI - NHC)	0.196	0.455	0.432	0.666	

Post-Op, post operation; CI, cochlear implant; NHC, normal hearing control.

Supplementary Table 7 A cumulative link mixed model for examining the effect of CI and condition between normal hearing controls' and the CI ears' subjective comprehension ratings:

rating ~ condition * group + (1|item) + (1|subject).

CI ear vs. normal hearing control					
**Post-Op 3-mon**					
**Regression weights**	**Estimate**	**Std. Err.**	**z value**	***P***	
condition difficult	0.263	0.240	1.095	0.273	
condition easy	1.180	0.232	5.083	<0.001	***
condition very easy	1.709	0.231	7.416	<0.001	***
group NHC	2.280	0.821	2.777	0.005	**
condition difficult:group NHC	0.442	0.228	1.937	0.053	.
condition easy:group NHC	0.717	0.219	3.270	0.001	**
condition very easy:group NHC	1.664	0.221	7.549	<0.001	***
reference category: condition very difficult, group CI ear					
**Comparisons of Group for each Condition**	**Estimate**	**Std. Err.**	**z value**	***P***	
Condition = very difficult (CI - NHC)	-2.280	0.821	-2.777	0.006	**
Condition = difficult (CI - NHC)	-2.720	0.819	-3.322	<0.001	***
Condition = easy (CI - NHC)	-3.000	0.816	-3.672	<0.000	***
Condition = very easy (CI - NHC)	-3.940	0.816	-4.833	<0.001	***
**Post-Op 6-mon**					
**Regression weights**	**Estimate**	**Std. Err.**	**z value**	***P***	
condition difficult	0.295	0.244	1.207	0.227	
condition easy	1.171	0.240	4.885	<0.001	***
condition very easy	1.694	0.240	7.067	<0.001	***
group NHC	1.802	0.828	2.176	0.030	*
condition difficult:group NHC	0.402	0.223	1.802	0.072	
condition easy:group NHC	0.704	0.217	3.251	0.001	**
condition very easy:group NHC	1.621	0.219	7.406	<0.001	***
reference category: condition very difficult, group CI ear					
**Comparisons of Group for each Condition**	**Estimate**	**Std. Err.**	**z value**	***P***	
Condition = very difficult (non-CI - NHC)	-1.800	0.828	-2.1760	0.030	*
Condition = difficult (non-CI - NHC)	-2.200	0.827	-2.6650	0.008	**
Condition = easy (non-CI - NHC)	-2.510	0.824	-3.0400	0.002	**

Condition = very easy (non-CI - NHC)	-3.420	0.824	-4.1520	<0.001	***

Post-Op 12-mon

Regression weights	Estimate	Std. Err.	z value	*P*	
condition difficult	0.342	0.235	1.457	0.145	
condition easy	1.333	0.230	5.791	<0.001	***
condition very easy	1.743	0.230	7.585	<0.001	***
group NHC	1.483	0.690	2.15	0.032	*
condition difficult:group NHC	0.306	0.212	1.445	0.149	
condition easy:group NHC	0.389	0.205	1.893	0.058	
condition very easy:group NHC	1.345	0.207	6.487	<0.001	***
reference category: condition very difficult, group CI ear					

Comparisons of Group for each Condition	Estimate	Std. Err.	z value	*P*	
Condition = very difficult (non-CI - NHC)	-1.480	0.690	-2.150	0.032	*
Condition = difficult (non-CI - NHC)	-1.790	0.687	-2.602	0.009	**
Condition = easy (non-CI - NHC)	-1.870	0.685	-2.733	0.006	**
Condition = very easy (non-CI - NHC)	-2.830	0.685	-4.126	<0.001	***

Post-Op, post operation; CI, cochlear implant; NHC, normal hearing control.

Supplementary Table 8 A cumulative link mixed model for examining the effect of condition, session and laterality in non-CI ears: rating ~ laterality*session*condition + (1|item) + (1|subject).

non-CI ear					
**Regression weights**	**Estimate**	**Std. Err.**	**z value**	***P***	
right ear	-0.256	0.523	-0.489	0.625	
post-op 3-mon	-0.228	0.299	-0.760	0.447	
post-op 6-mon	1.094	0.252	4.341	<0.001	***
post-op 12-mon	0.775	0.257	3.020	0.003	**
condition difficult	1.057	0.328	3.227	0.001	**
condition easy	2.220	0.320	6.945	<0.001	***
condition very easy	3.683	0.316	11.644	<0.001	***
right ear:post-op 3-mon	0.245	0.420	0.584	0.559	
right ear:post-op 6-mon	0.466	0.356	1.309	0.191	
right ear:post-op 12-mon	0.197	0.367	0.535	0.593	
right ear:condition difficult	-0.043	0.363	-0.120	0.905	
right ear:condition easy	0.437	0.343	1.275	0.202	
right ear:condition very easy	0.624	0.337	1.854	0.064	.
post-op 3-mon:condition difficult	0.292	0.372	0.785	0.433	
post-op 6-mon:condition difficult	-0.609	0.329	-1.852	0.064	
post-op 12-mon:condition difficult	0.044	0.329	0.133	0.894	
post-op 3-mon:condition easy	-0.044	0.357	-0.122	0.903	
post-op 6-mon:condition easy	-0.598	0.315	-1.900	0.057	
post-op 12-mon:condition easy	-0.330	0.319	-1.035	0.301	
post-op 3-mon:condition very easy	0.219	0.347	0.633	0.527	
post-op 6-mon:condition very easy	-0.257	0.306	-0.839	0.402	
post-op 12-mon:condition very easy	0.245	0.311	0.787	0.431	
right ear:post-op 3-mon:condition difficult	0.015	0.523	0.029	0.977	
right ear:post-op 6-mon:condition difficult	0.667	0.461	1.447	0.148	
right ear:post-op 12-mon:condition difficult	0.020	0.469	0.044	0.965	
right ear:post-op 3-mon:condition easy	0.123	0.496	0.248	0.804	
right ear:post-op 6-mon:condition easy	0.222	0.439	0.506	0.613	

right ear:post-op 12-mon:condition easy	0.346	0.449	0.771	0.441
right ear:post-op 3-mon:condition very easy	-0.149	0.486	-0.307	0.759
right ear:post-op 6-mon:condition very easy	0.015	0.433	0.035	0.972
right ear:post-op 12-mon:condition very easy	0.027	0.443	0.061	0.951

reference category: left ear, pre-op 3, condition very difficult

**Analysis of Deviance**	**LR Chisq**	**Df**	***P***	
laterality	0.76	1	0.3834	
session	393.89	3	<0.001	***
condition	209.6	3	<0.001	***
laterality:session	26.15	3	<0.001	***
laterality:condition	27.69	3	<0.001	***
session:condition	15.33	9	0.0822	
laterality:session:condition	7.12	9	0.6246	

Post-Op, post operation; CI, cochlear implant.

Supplementary Table 9 A cumulative link mixed model for examining the effect of condition, session and laterality in CI ears:
rating ~ laterality*session*condition + (1|item) + (1|subject).

CI ear					
**Regression weights**	**Estimate**	**Std. Err.**	**z value**	***P***	
right ear	0.119	1.335	0.089	0.929	
post-op 6-mon	0.018	0.228	0.078	0.938	
post-op 12-mon	-0.624	0.248	-2.513	0.012	*
condition difficult	0.347	0.254	1.368	0.171	
condition easy	0.646	0.250	2.586	0.010	**
condition very easy	1.133	0.245	4.626	0.000	***
right ear:post-op 6-mon	0.903	0.352	2.566	0.010	*
right ear:post-op 12-mon	1.798	0.360	4.992	0.000	***
right ear:condition difficult	-0.255	0.370	-0.691	0.490	
right ear:condition easy	0.816	0.338	2.414	0.016	*
right ear:condition very easy	0.680	0.331	2.057	0.040	*
post-op 6-mon:condition difficult	0.057	0.313	0.182	0.856	
post-op 12-mon:condition difficult	-0.140	0.343	-0.407	0.684	
post-op 6-mon:condition easy	0.424	0.306	1.387	0.165	
post-op 12-mon:condition easy	0.619	0.330	1.875	0.061	.
post-op 6-mon:condition very easy	0.182	0.301	0.607	0.544	
post-op 12-mon:condition very easy	0.160	0.326	0.490	0.624	
right ear:post-op 6-mon:condition difficult	-0.055	0.488	-0.113	0.910	
right ear:post-op 12-mon:condition difficult	0.492	0.498	0.989	0.323	
right ear:post-op 6-mon:condition easy	-1.117	0.456	-2.448	0.014	*
right ear:post-op 12-mon:condition easy	-0.745	0.464	-1.606	0.108	
right ear:post-op 6-mon:condition very easy	-0.688	0.448	-1.535	0.125	
right ear:post-op 12-mon:condition very easy	-0.089	0.458	-0.195	0.845	
reference category: post-op 3-mon, condition very difficult					

**Analysis of Deviance**	**LR Chisq**	**Df**	***P***	
laterality	0.832	1	0.362	
session	37.958	2	<0.001	***

condition	98.542	3	<0.001	***
laterality:session	125.798	2	<0.001	***
laterality:condition	9.389	3	0.025	*
session:condition	3.595	6	0.731	
laterality:session:condition	12.321	6	0.055	

Post-Op, post operation; CI, cochlear implant.

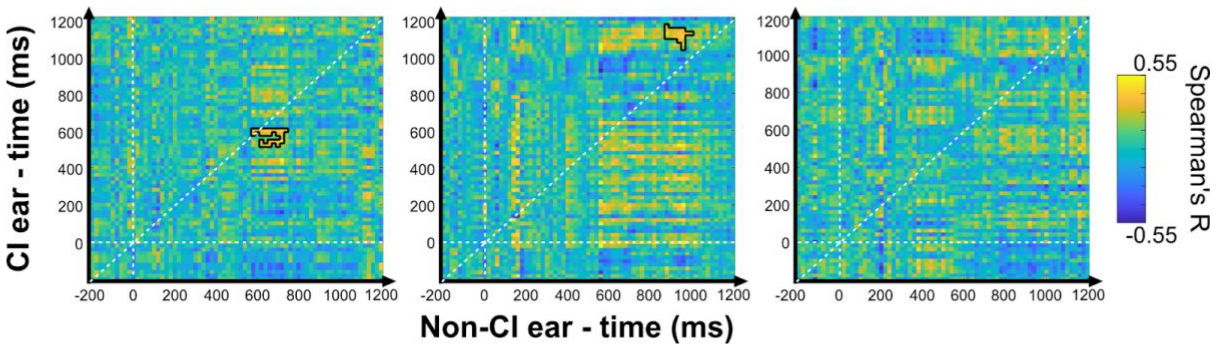

Supplementary Figure 5 Quantification of shared degraded speech representation between CI users' CI ear and non-CI ear. Average time-generalized Spearman's R matrices relating CI ears and non-CI ears for each session. Significant correlations are highlighted with black lines (N = 10 for each group, right-tailed sign permutation tests, cluster-corrected significance level $P < 0.05$). It's important to note that comparing ears within CI subjects is problematic due to the lack of a clear and healthy reference. The assumption that the non-implanted ear of a CI subject functions normally is difficult to verify.

Supplementary Reference

1. Obleser J, Weisz N. Suppressed alpha oscillations predict intelligibility of speech and its acoustic details. *Cereb Cortex*. 2012;22(11):2466-2477. doi:10.1093/cercor/bhr325
